# Supplementary material for: Evolution of larval segment position across 12 Drosophila species
Source: Evolution. 2020 Jan 20;74(7):1409–22. doi: 10.1111/evo.13911 (PMC7496318; doi:10.1111/evo.13911)
Supplement: Supplementary file 1 — Figure S1. Testing the effect of larval mounting procedures on relative segment position. [file EVO-74-1409-s006.docx]

**Figure S1.** Testing the effect of larval mounting procedures on relative segment position. For this experiment, relative segment position measurements were taken before and after multiple stages of the mounting protocol, to test the effect of the mounting protocol on segment position across multiple species (see Methods) (A) Bright field image of a *D. melanogaster* first instar larva, hatched in water, iced for 2-3 minutes before imaging. (B) The same *D. melanogaster* larva heated at 60°C for an hour prior to bright field imaging. (C) Dark field image of the same *D. melanogaster* larva, mounted using PVA, and incubated in the 60°C oven overnight. This represents the end stage of our mounting protocol. White horizontal lines mark the full length of the larvae, vertical lines mark the segment borders.
