# Supplementary material for: Evolution of larval segment position across 12 Drosophila species
Source: Evolution. 2020 Jan 20;74(7):1409–22. doi: 10.1111/evo.13911 (PMC7496318; doi:10.1111/evo.13911)
Supplement: Supplementary file 2 — Figure S2. Comparing pairs of species, most segments were in different relative positions. [file EVO-74-1409-s007.docx]

**Figure S2.** Comparing pairs of species, most segments were in different relative positions. For the majority of species pair comparisons, five or more segments had significantly shifted relative positions (t-tests with Bonferroni correction, p-value ≤ 0.05). The graph shows the number of species pairs on the y-axis and number of segments significantly shifted (see Methods) on the x-axis.
