# Supplementary material for: Evolution of larval segment position across 12 Drosophila species
Source: Evolution. 2020 Jan 20;74(7):1409–22. doi: 10.1111/evo.13911 (PMC7496318; doi:10.1111/evo.13911)
Supplement: Supplementary file 3 — Figure S3. The posterior‐most segments show the largest magnitude in differences from mean across species. [file EVO-74-1409-s008.docx]

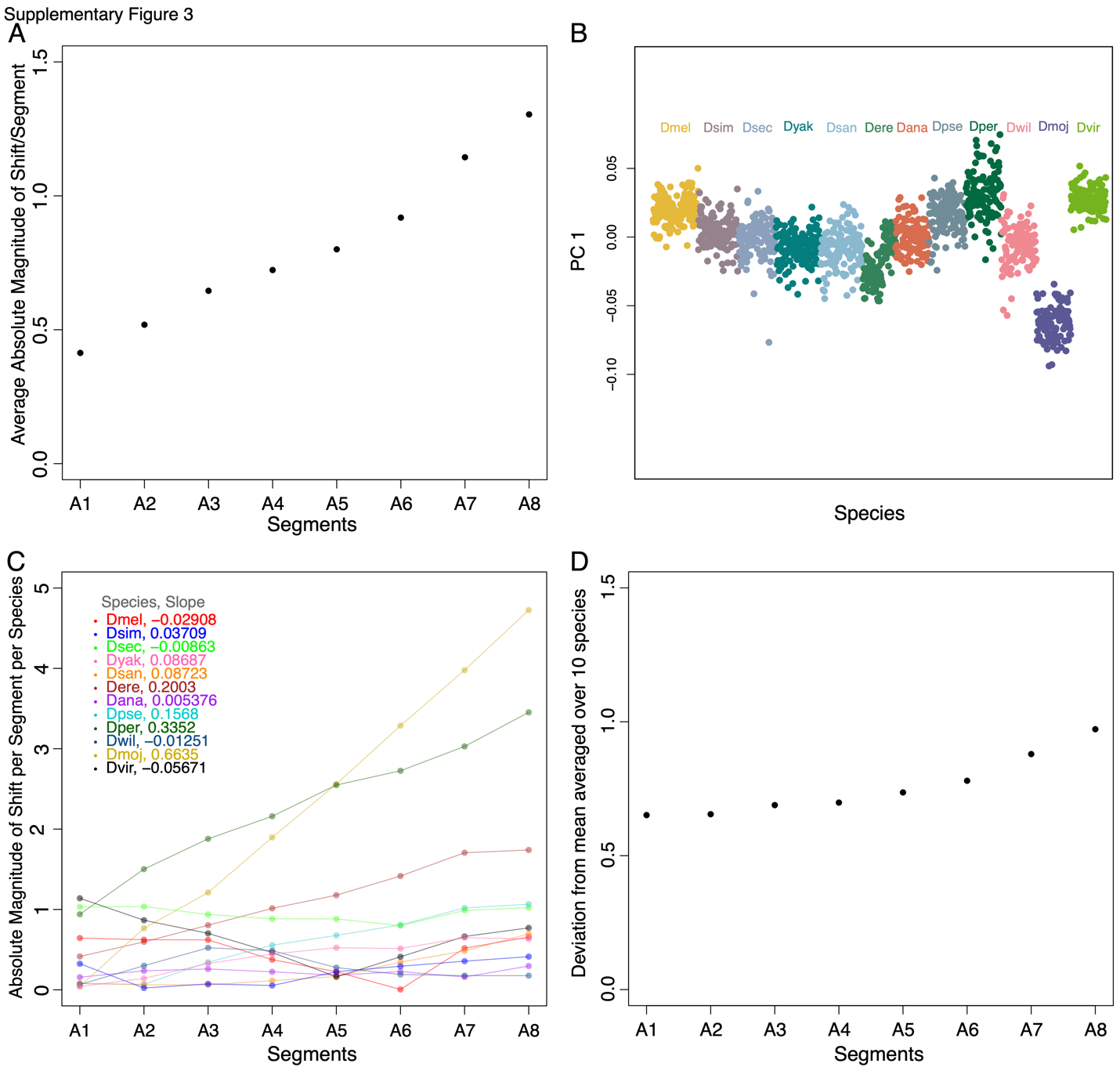


**Figure S3.** The posterior-most segments show the largest magnitude in differences from mean across species. (A) Average absolute magnitude of shift in the relative position of each segment, as compared to the across species mean, increases from anterior to the posterior of the larvae. On the y-axis of this graph is absolute magnitude of shift, in percent larval length, for each segment averaged over all species, on the x-axis are abdominal segments A1 through A8. (B) From principal component analysis, PC1 is plotted for all species. Positive values correspond to posterior shifts of segments, negative values indicate anterior shifts of segments, relative to mean centered positions for each segment. PC1 explains 88% of the variance and is highly positively correlated with the positions of the most posterior segments (Pearson correlation coefficients with each segment:  A1= -0.15, A2=0.11, A3=0.28, A4=0.49, A5=0.64, A6=0.77, A7=0.85, A8=0.88). Note the similarity in pattern to Figure 3, with *D. mojavensis* having the largest deviation from the rest of the species, with a dramatic shift toward the anterior (negative PC1 values) and *D. persimilis* shifted toward the posterior (positive PC1 values). (C) Absolute magnitude of shift changes from anterior to posterior differently for each of the 12 *Drosophila* species. The graph shows absolute magnitude of shift in percent larval length on the y-axis and abdominal segments A1 through A8 on the x-axis. The legend in the graph shows the slopes calculated for each trend form anterior to posterior. Lines in the graph and the legend are color coordinated for each species. (D) Average absolute magnitude of shift in the relative position of each segment, as compared to the across species mean, increases from anterior to the posterior of the larvae even in the absence of segment position data for *D. persimilis* and *D. mojavensis* (compare to Figure S3A, this representation is the same, except with *D. persimilis* and *D. mojavensis* removed from the calculation of the mean). On the y-axis of this graph is absolute magnitude of shift, in percent larval length, for each segment averaged over all species, on the x-axis are abdominal segments A1 through A8.
