# Supplementary material for: Evolution of larval segment position across 12 Drosophila species
Source: Evolution. 2020 Jan 20;74(7):1409–22. doi: 10.1111/evo.13911 (PMC7496318; doi:10.1111/evo.13911)
Supplement: Supplementary file 4 — Figure S4. The A8+tail region is responsible for much of the total amount (both number and magnitude) of significant differences between species. [file EVO-74-1409-s009.docx]

**Figure S4.** The A8+tail region is responsible for much of the total amount (both number and magnitude) of significant differences between species. For all these plots, compare the effect of when A8+tail is removed to when another terminal segment (h+t) is removed, to determine whether the effect is A8+tail specific or simply an effect of removing a terminal segment. (A) Comparison of numbers of significant position changes when segment positions were calculated with all segments included, with h+t removed or with A8+tail removed. Removal of A8+tail shows a decrease, whereas removal of h+t shows an increase, in the number of segments that have significantly changed their relative position as compared to when all eight abdominal segments were included in the position calculations. Y-axis is total number of significant changes, normalized to the number of segments included in position calculations. (B) This plot shows the same data as part A, but illustrates how the number of significant position changes varies across the length of the larva. When A8+tail was removed from position calculations, number of significant changes in relative segment position decreased sharply towards the posterior end of the larvae. However, when h+t was removed in the same manner, number of significant changes in relative segment position followed the same pattern from anterior to posterior as when all abdominal segments were included in position calculations. y-axis is number of significant changes in relative position normalized to the number of segments included in position calculations. (C) Average absolute magnitude of shift (from the species mean) for each segment decreased sharply towards the posterior of the larvae when A8+tail was removed from segment position calculations. On the other hand, removing h+t followed the same pattern as when all segments were included in calculations, but showed a bigger deviation from the species mean towards the posterior of the larvae. y-axis is the average magnitude of shift (from the species mean) in segment position, in percent larval length, ignoring the direction. X-axis is abdominal segments in both panels B and C.
