# Supplementary material for: Evolution of larval segment position across 12 Drosophila species
Source: Evolution. 2020 Jan 20;74(7):1409–22. doi: 10.1111/evo.13911 (PMC7496318; doi:10.1111/evo.13911)
Supplement: Supplementary file 5 — Figure S5. Features of body length. [file EVO-74-1409-s010.docx]

**
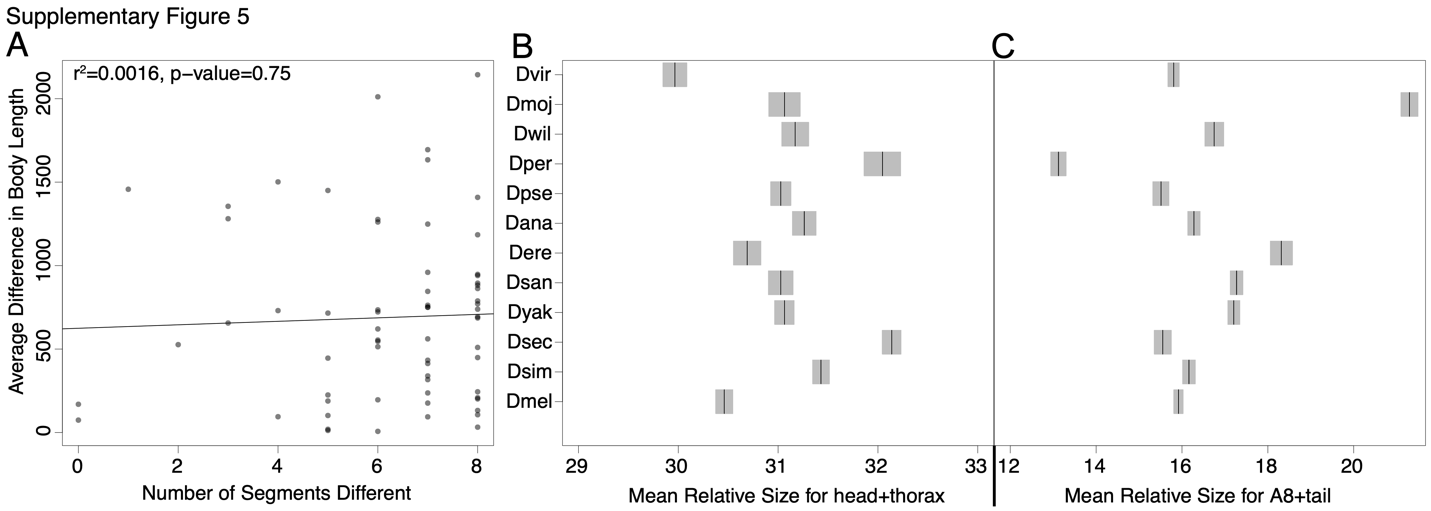
**

**Figure S5.** Features of body length. (A) Average body length difference between a given pair of species is not correlated with the number of segments with significant position shifts between the species. The y-axis in this graph shows average difference in body length (in pixels) between each pair of species and the x-axis shows the number of segments with significant position shifts in the same species pair. (B) This graph shows differences in mean relative size for h+t, in percent larval length, among 12 *Drosophila* species. The black bars indicate the mean and the gray shaded area is 95% confidence interval for each mean. (C) This graph shows differences in mean relative size for A8+tail, in percent larval length, among 12 *Drosophila* species. The black bars indicate the mean and the gray shaded area is 95% confidence interval for each mean. Parts B and C represent transformations of the position and length measurements, to highlight the length of the terminal portions of the larva.
