# Supplementary material for: Evolution of larval segment position across 12 Drosophila species
Source: Evolution. 2020 Jan 20;74(7):1409–22. doi: 10.1111/evo.13911 (PMC7496318; doi:10.1111/evo.13911)
Supplement: Supplementary file 6 — Figure S6. Coefficient of variation in segment positioning stays low throughout the larva, indicating that the relative positions of all segments are equally as precisely determined. However, standard deviation of segment positioning increases towards the posterior end of the larva, as the measurement values increase (relative position is measured in % of larval length from the anterior end). [file EVO-74-1409-s011.docx]

**Figure S6.** Coefficient of variation in segment positioning stays low throughout the larva, indicating that the relative positions of all segments are equally as precisely determined. However, standard deviation of segment positioning increases towards the posterior end of the larva, as the measurement values increase (relative position is measured in % of larval length from the anterior end). (A) Change in coefficient of variation, averaged over 12 *Drosophila* species, for all segments from anterior to posterior of the larva. The y-axis shows coefficient of variation, and the x-axis depicts each abdominal segment. When segment positions were calculated with all segments included (black dots), coefficient of variation (noise) did not change along the anterior-posterior axis of the larva, and remained low. Removal of A8+tail (red dots) overall decreased noise levels in segment positioning, whereas removal of h+t (blue dots) increased noise levels towards the posterior of the larva. (B) Change in standard deviation, averaged over 12 species, for all segments from anterior to posterior of the larva. The y-axis shows standard deviation and the x-axis depicts each abdominal segment. When segment positions were calculated with all segments included (black dots), standard deviation of segment positions increased towards the posterior of the larva. Removal of A8+tail (red dots) decreased standard deviations in the posterior, whereas removal of h+t (blue dots) increased standard deviations towards the posterior of the larva.
