# Supplementary material for: Evolution of larval segment position across 12 Drosophila species
Source: Evolution. 2020 Jan 20;74(7):1409–22. doi: 10.1111/evo.13911 (PMC7496318; doi:10.1111/evo.13911)
Supplement: Supplementary file 7 — Figure S7. Relative segment position and body size is highly variable among 12 species of Drosophila, although less so when A8+ tail is removed. [file EVO-74-1409-s012.docx]

**Figure S7.** Relative segment position and body size is highly variable among 12 species of *Drosophila,* though less so when A8+ tail is removed. These graphs have the same format as Figure 2B and shows differences in relative segment position between species. (A) Relative segment positions are calculated in the absence of A8+tail. Notice a substantial number of nonsignificant differences as compared to Figure 2B. (B) Relative segment positions are calculated in the absence of h+t and also with measurements taken from the posterior of the larva instead of the anterior. As compared to Figure 2B the segment order is flipped, but the pattern of change in segment position is very similar. In both graphs, each black bar depicts the mean for each segment in each species. The colored areas are 95% confidence intervals. The y-axis depicts 12 *Drosophila* species and x-axis shows mean relative abdominal segment position in percent larval length.
