# Supplementary material for: Evolution of larval segment position across 12 Drosophila species
Source: Evolution. 2020 Jan 20;74(7):1409–22. doi: 10.1111/evo.13911 (PMC7496318; doi:10.1111/evo.13911)
Supplement: Supplementary file 8 — Figure S8. Phylogenetic analysis of relative segment evolution in the 12 Drosophila species, with posterior rate estimates for all branches. [file EVO-74-1409-s013.docx]

**Figure S8.** Phylogenetic analysis of relative segment evolution in the 12 *Drosophila* species, with posterior rate estimates for all branches. A) Like Figure 4A, the phylogeny was inferred from nuclear loci with relative divergence times, and branches of the tree are colored to indicate the overall rate of relative segment position evolution. Here, the branches are numbered to correspond with the values pictured in part B. B) Box plot depicting rate of evolution estimate for each branch of the phylogeny, x-axis corresponds to the branch numbers indicated on the tree in part A.
