# Supplementary material for: Evolution of larval segment position across 12 Drosophila species
Source: Evolution. 2020 Jan 20;74(7):1409–22. doi: 10.1111/evo.13911 (PMC7496318; doi:10.1111/evo.13911)

**Figure S9.** Phylogenetic analysis of relative segment position for each segment. Like Figure 4A, these eight plots show a phylogeny inferred from nuclear loci with relative divergence times, and the branches of the tree are colored to indicate the overall rate of segment position evolution. Like Figure S8, the tree diagrams also include numbers for each linage, and are accompanied by a box plot showing the estimates for rates of segment evolution by each numbered lineage. The 8 plots show rate estimates for each abdominal segment separately.

Supplementary Figure 9

Segment 1

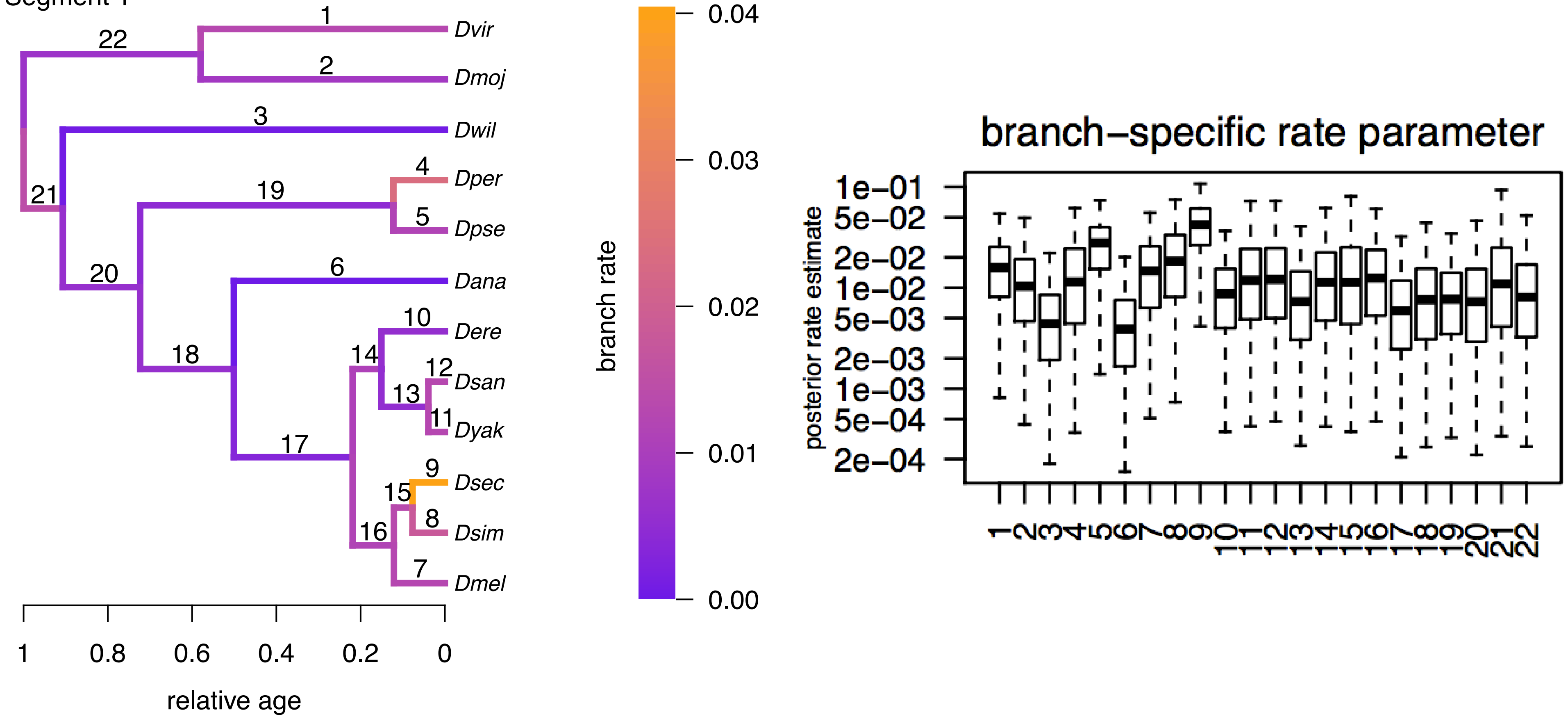

Supplementary Figure 9

Segment 2

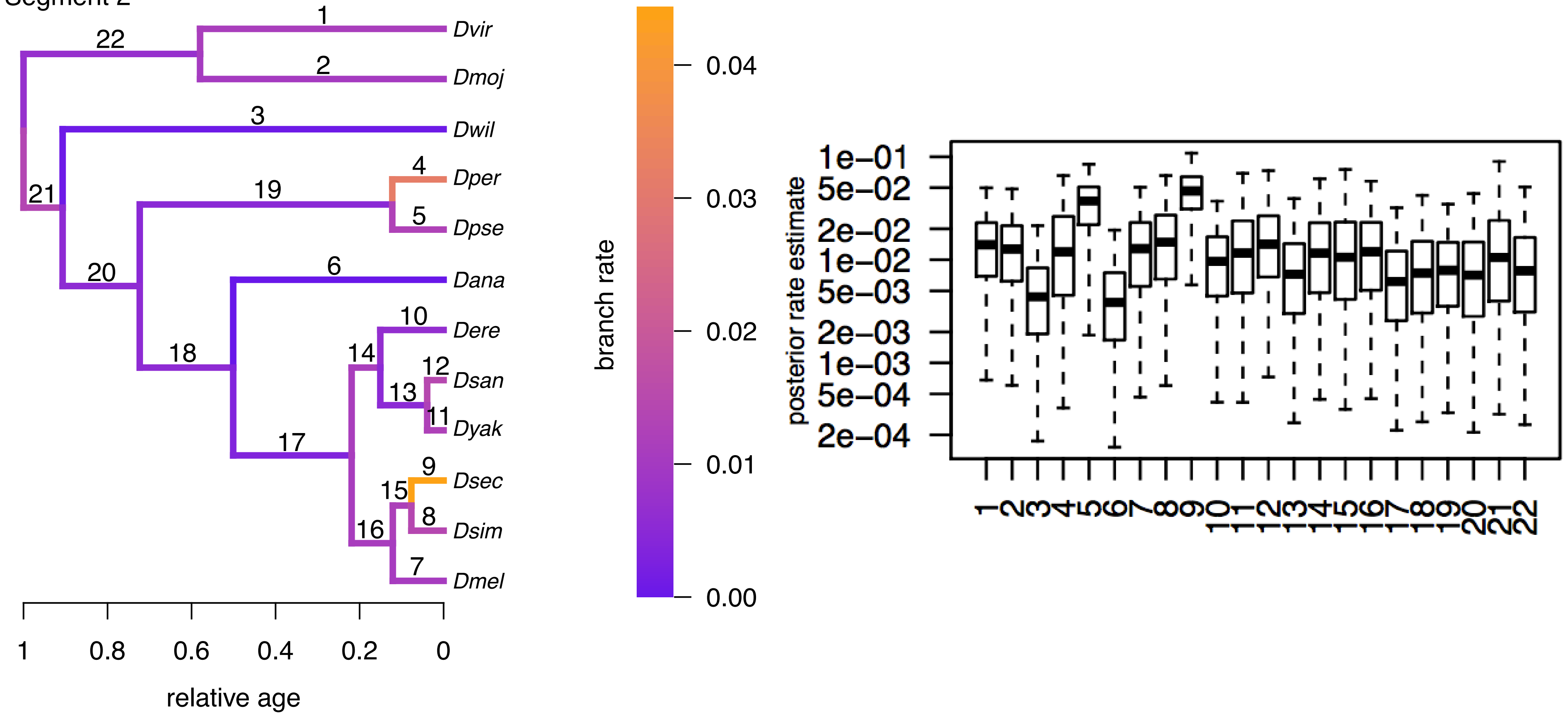

Supplementary Figure 9

Segment 3

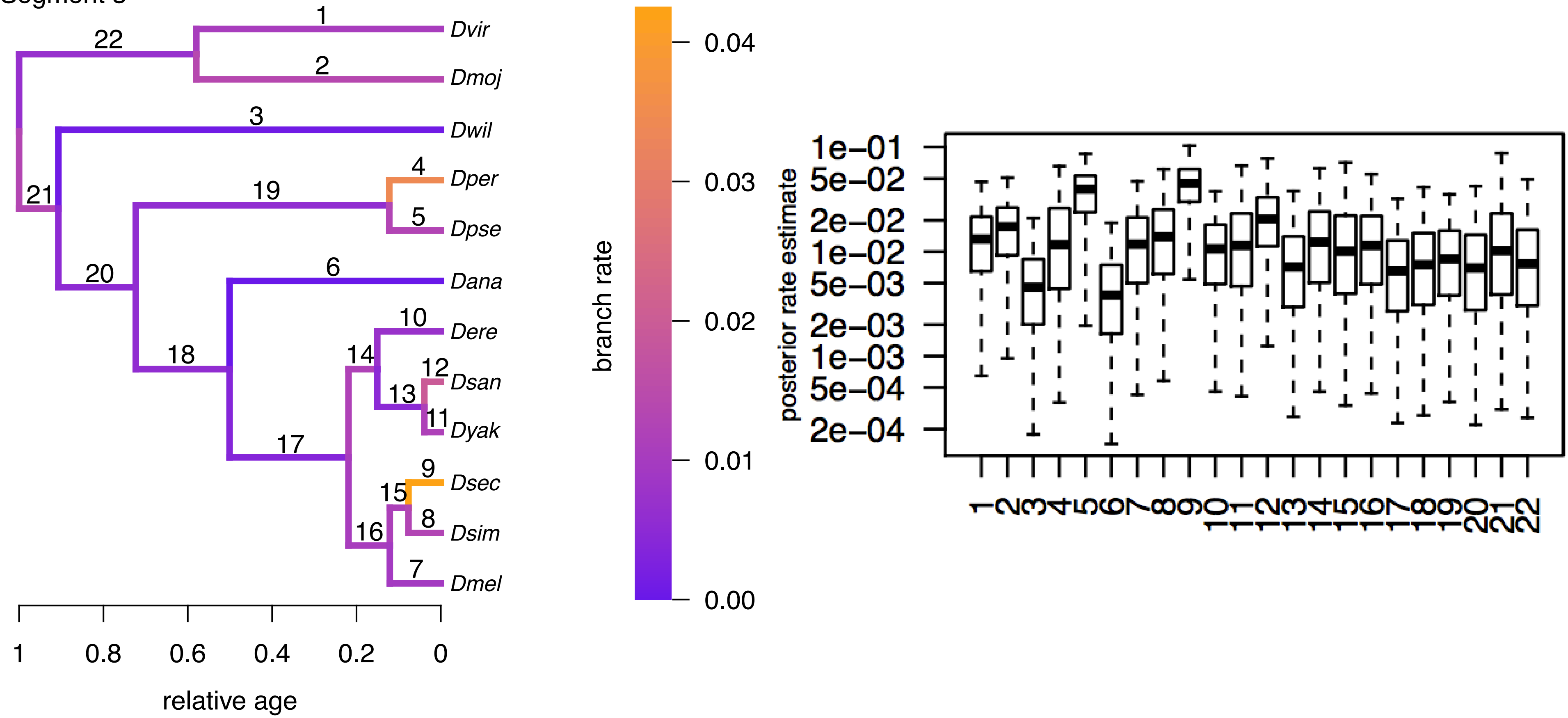

Supplementary Figure 9

Segment 4

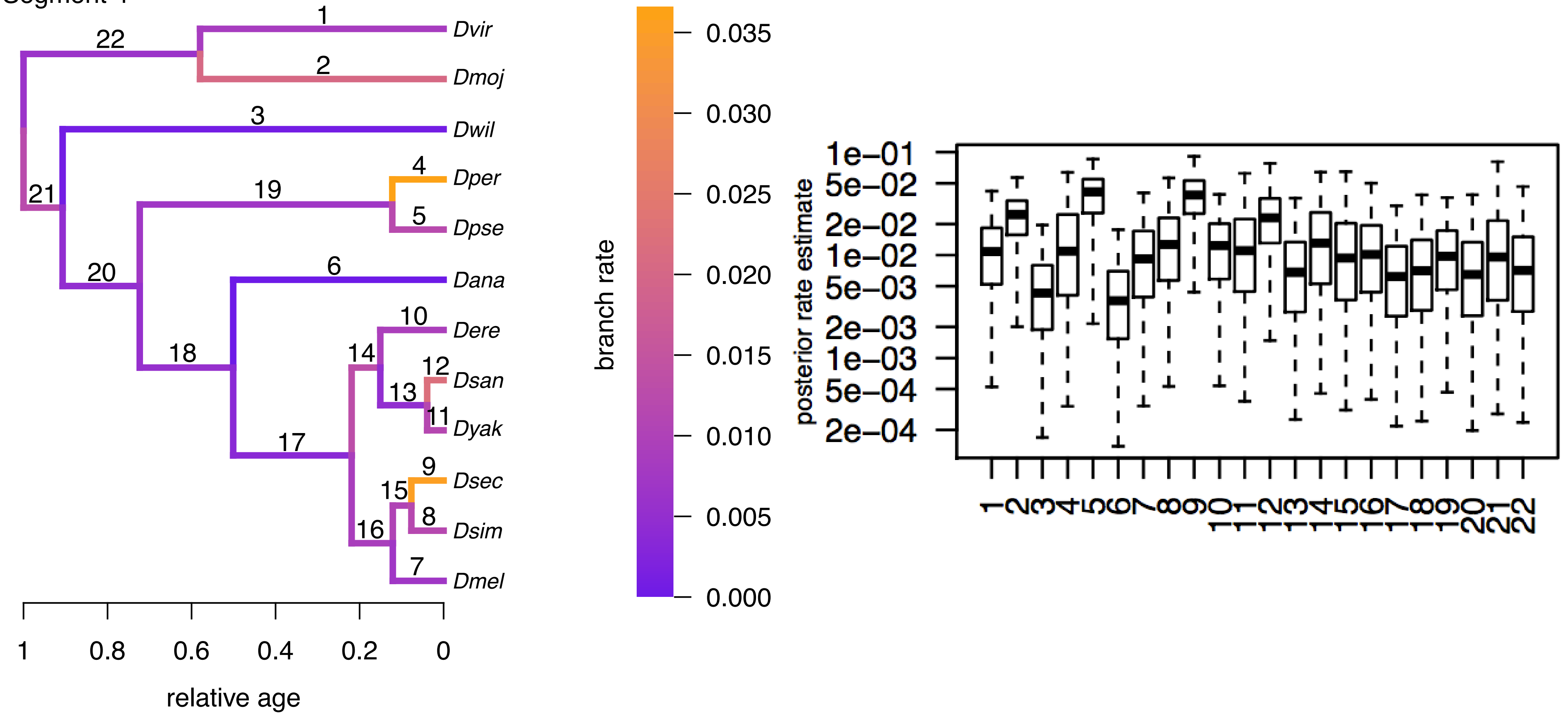

Supplementary Figure 9

Segment 5

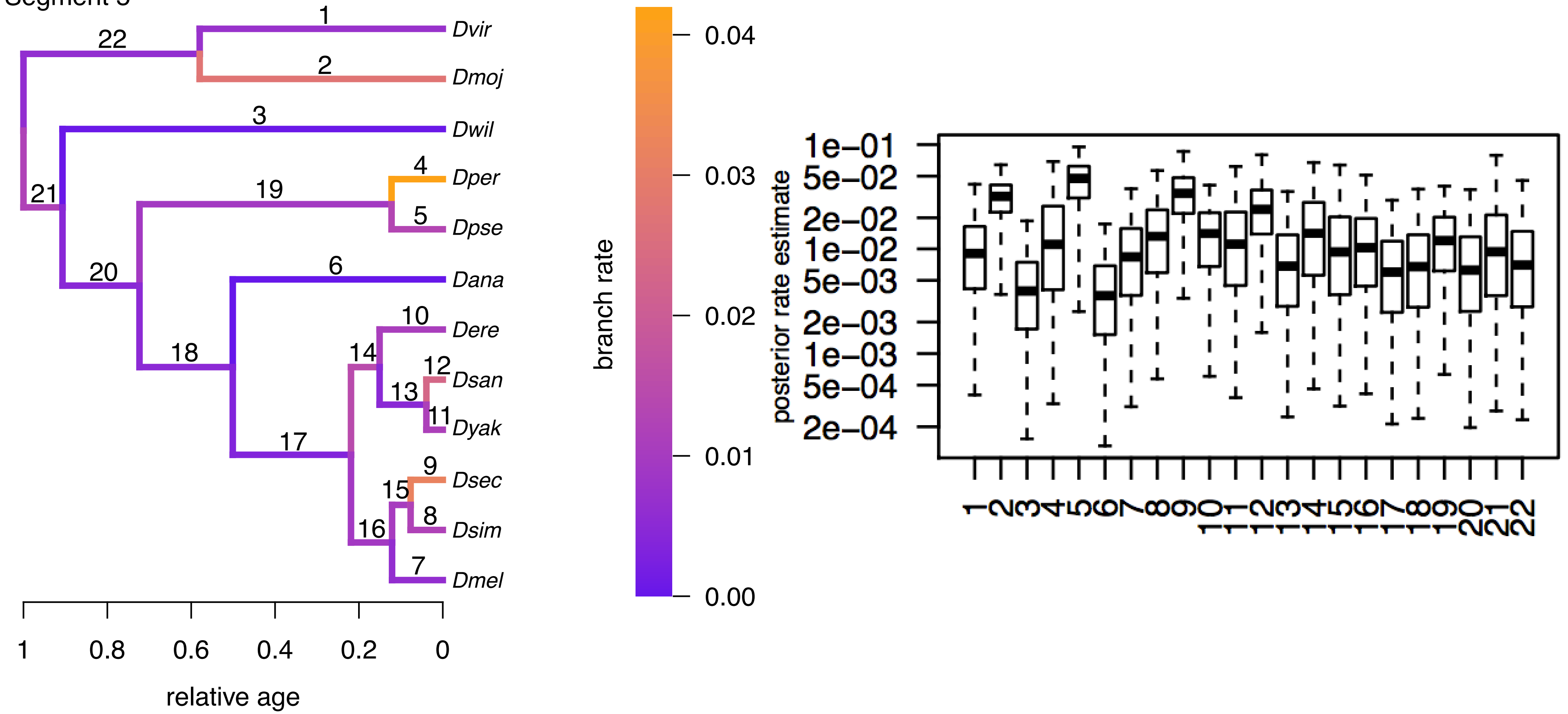

Supplementary Figure 9

Segment 6

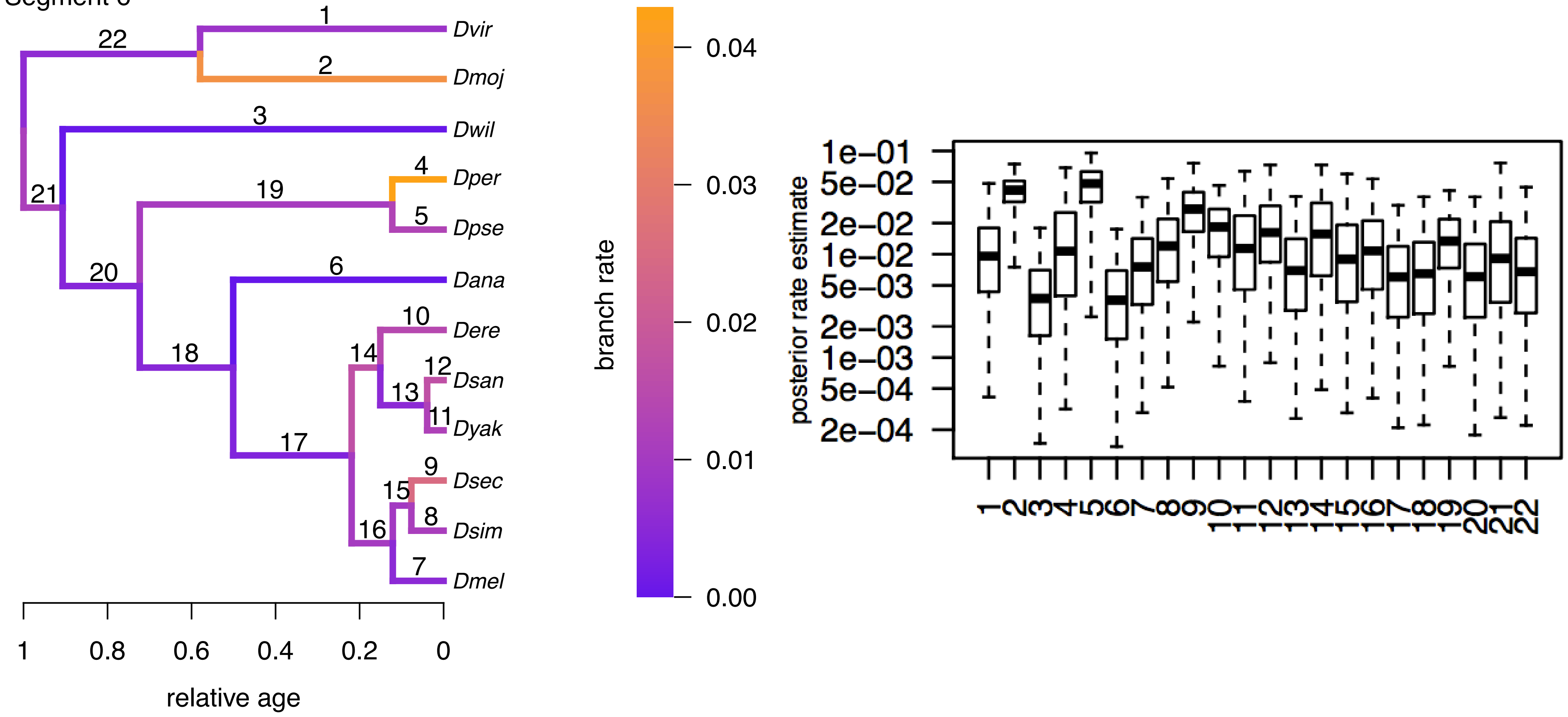

Supplementary Figure 9

Segment 7

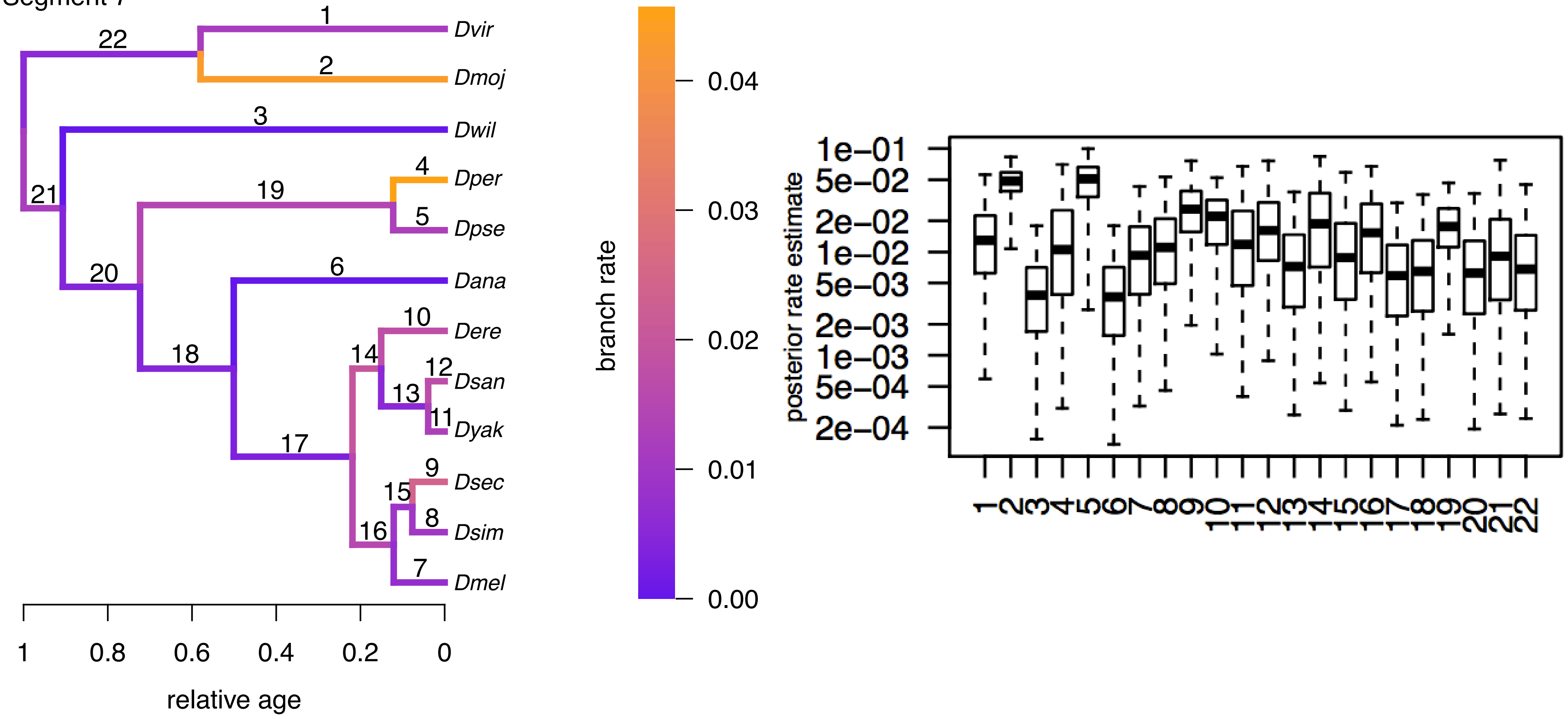

Supplementary Figure 9

Segment 8

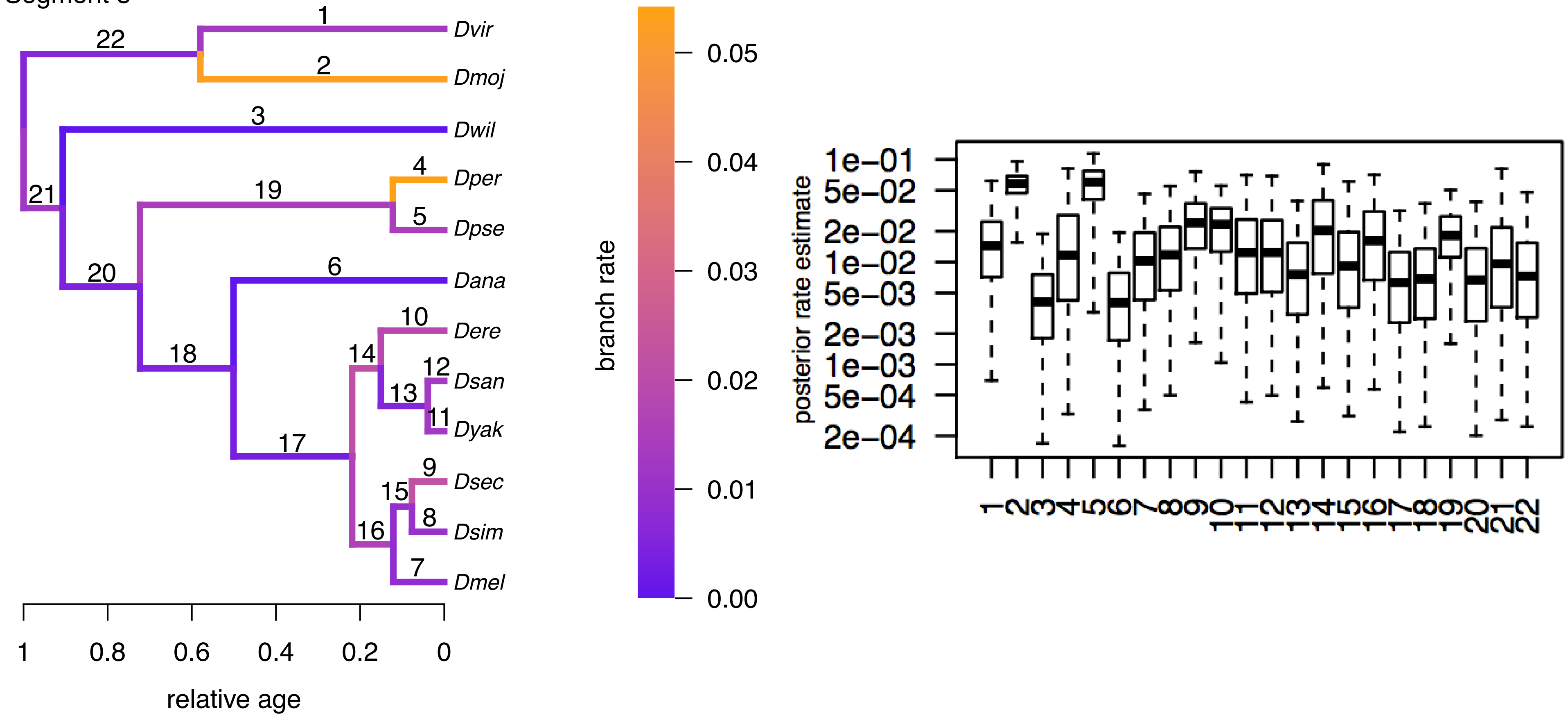

Supplement: Supplementary file 9 — Figure S9. Phylogenetic analysis of relative segment position for each segment. [file EVO-74-1409-s014.pdf]
