# Supplementary material for: Evolution of larval segment position across 12 Drosophila species
Source: Evolution. 2020 Jan 20;74(7):1409–22. doi: 10.1111/evo.13911 (PMC7496318; doi:10.1111/evo.13911)

Figure S10. Correlations between relative segment positions within each species. This series of graphs are constructed the same as Figure 5A, which was for *D. melanogaste*r, for each of the 12 *Drosophila* species. The y-axis shows correlation coefficients and the x-axis shows mean relative distance between pairs of segments in percent larval length.

Supplementary Figure 10

D.melanogaster Correlation score vs Pairwise distance

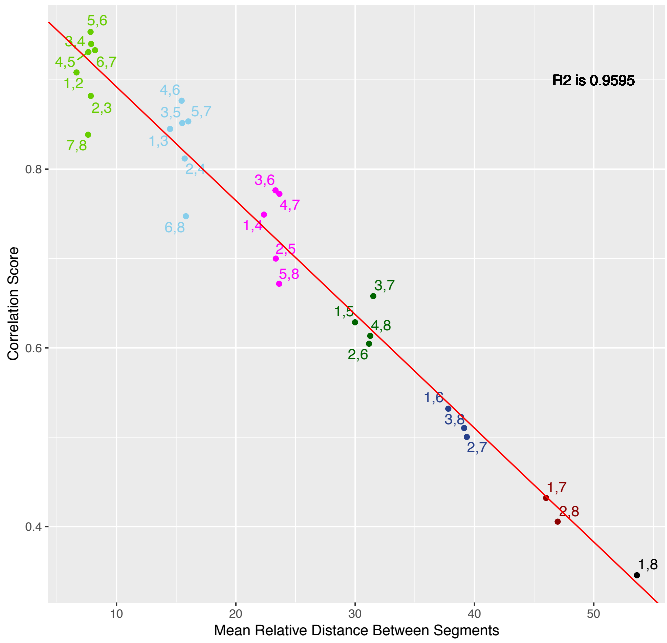

Supplementary Figure 10

D.simulans Correlation score vs Pairwise distance

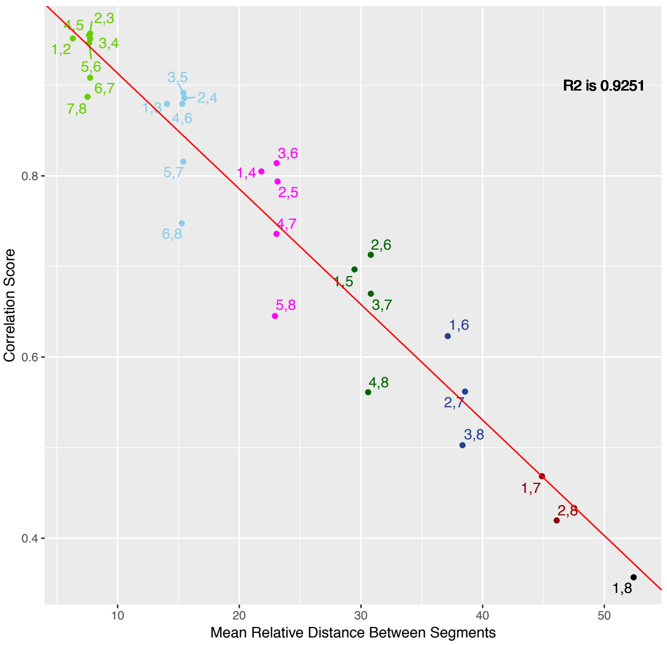

Supplementary Figure 10

D.sechellia Correlation score vs Pairwise distance

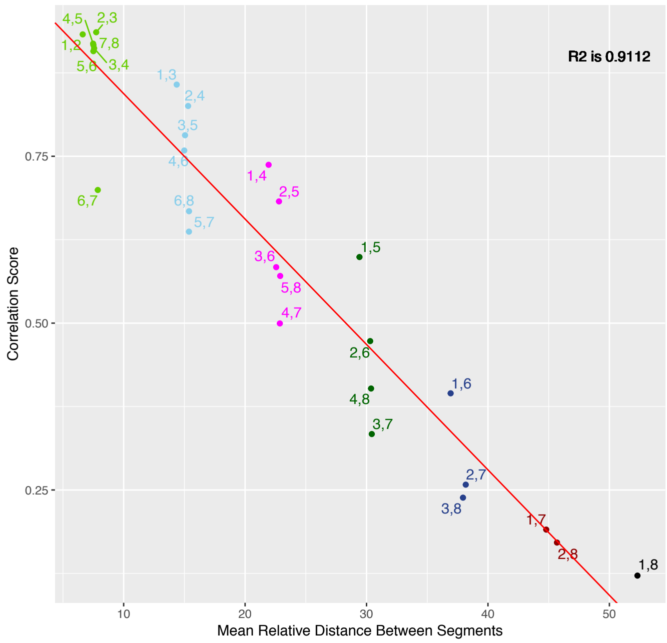

Supplementary Figure 10

D.yakuba Correlation score vs Pairwise distance

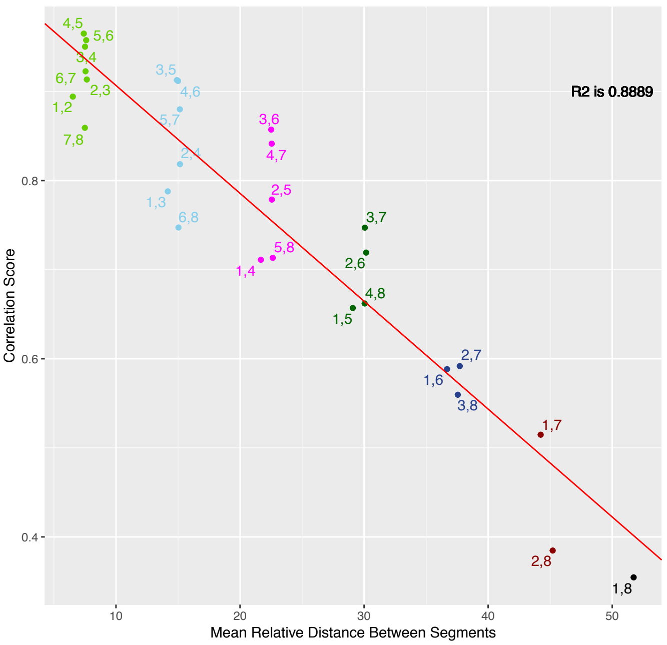

Supplementary Figure 10

D.santomea Correlation score vs Pairwise distance

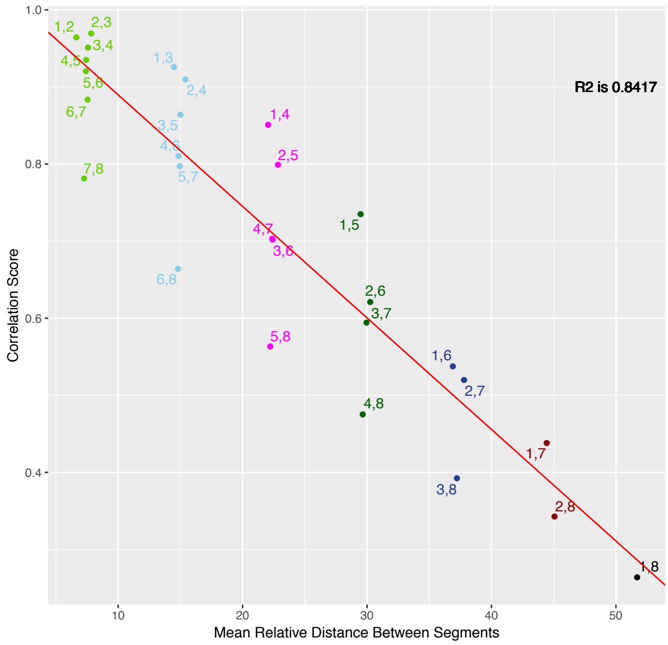

Supplementary Figure 10

D.erecta Correlation score vs Pairwise distance

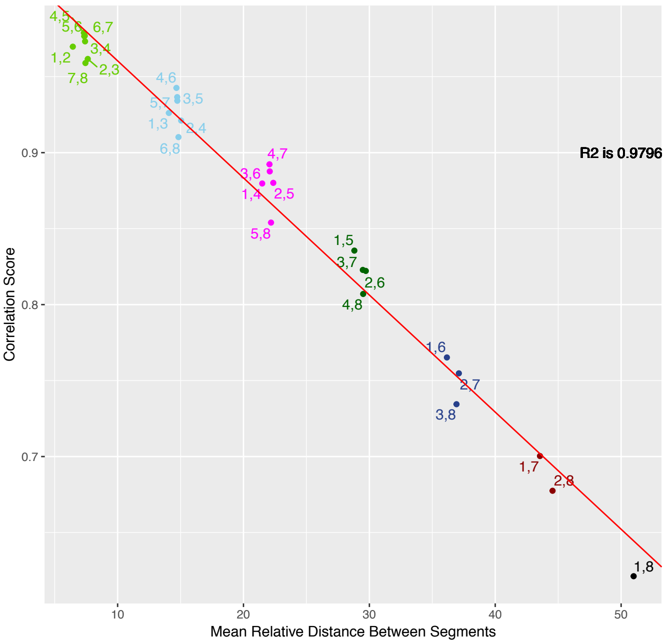

Supplementary Figure 10

D.ananassae Correlation score vs Pairwise distance

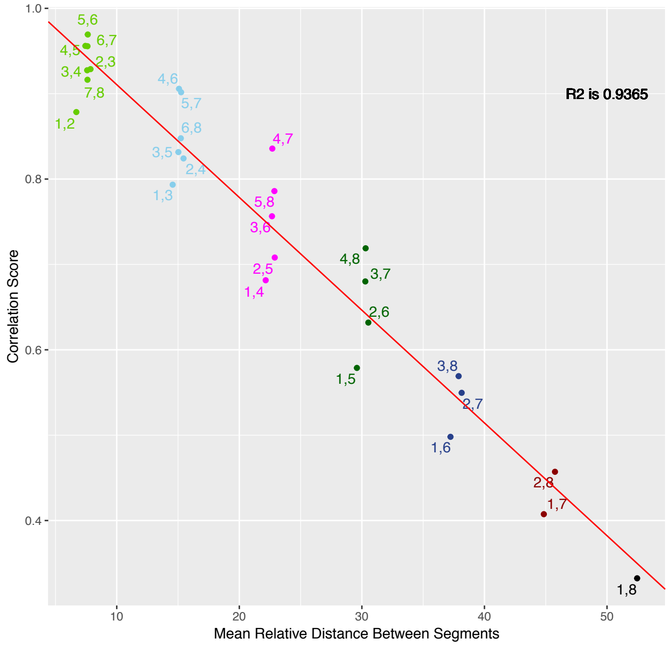

Supplementary Figure 10

D.pseudoobscura Correlation score vs Pairwise distance

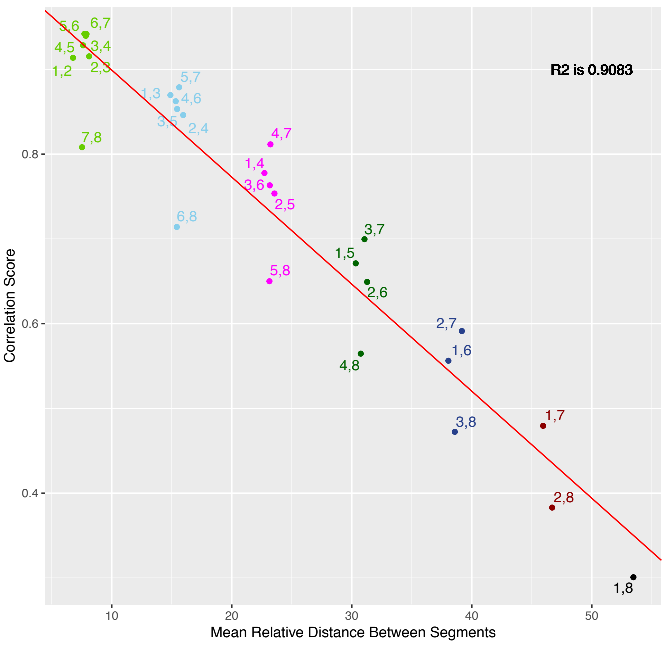

Supplementary Figure 10

D.persimilis Correlation score vs Pairwise distance

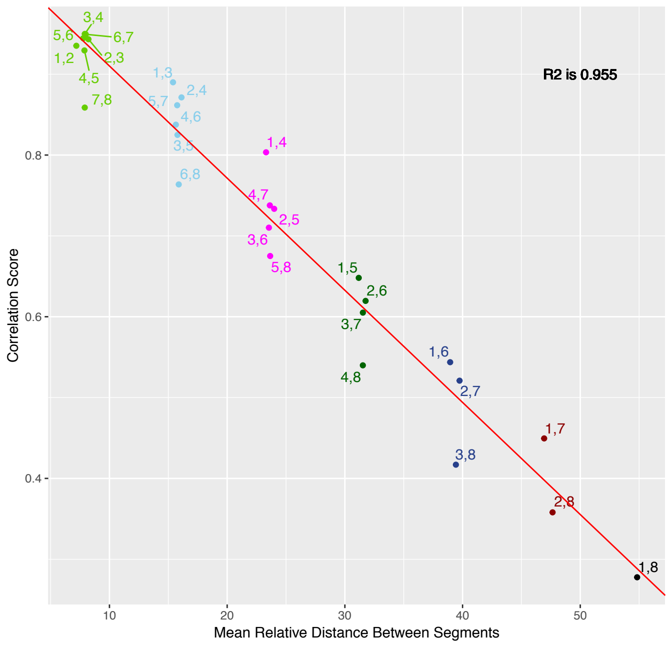

Supplementary Figure 10

D.willistoni Correlation score vs Pairwise distance

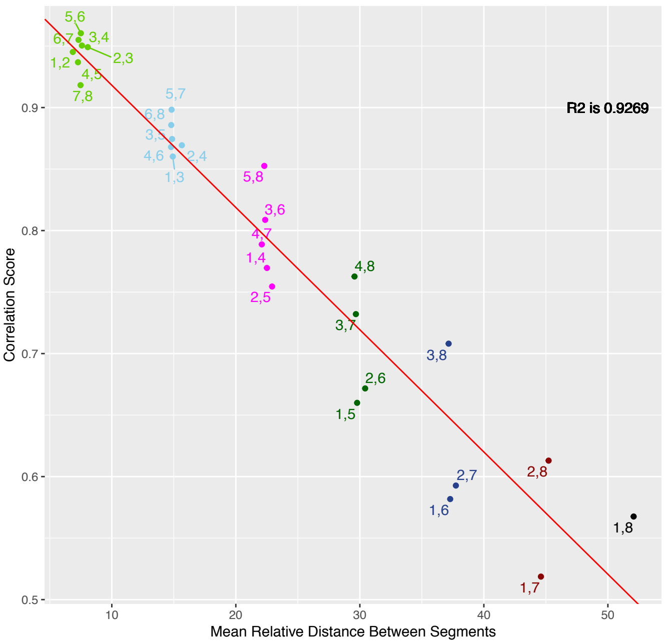

Supplementary Figure 10

D.mojavensis Correlation score vs Pairwise distance

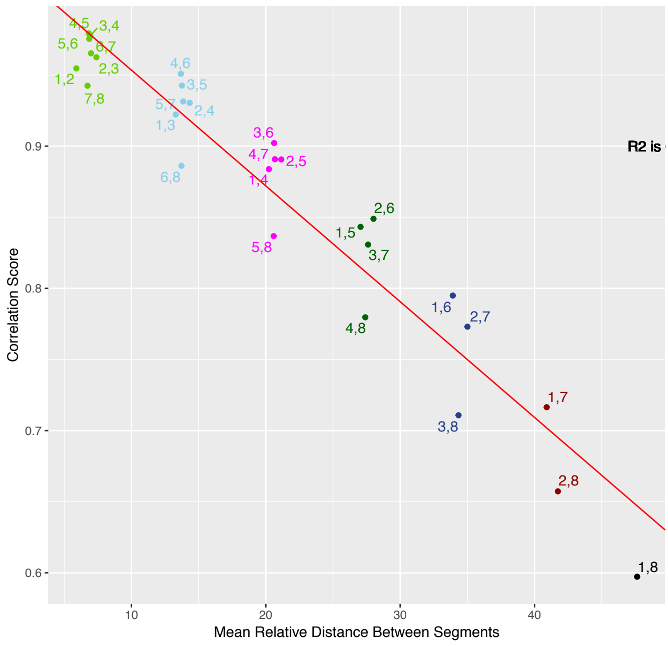

Supplementary Figure 10

D.virilis Correlation score vs Pairwise distance

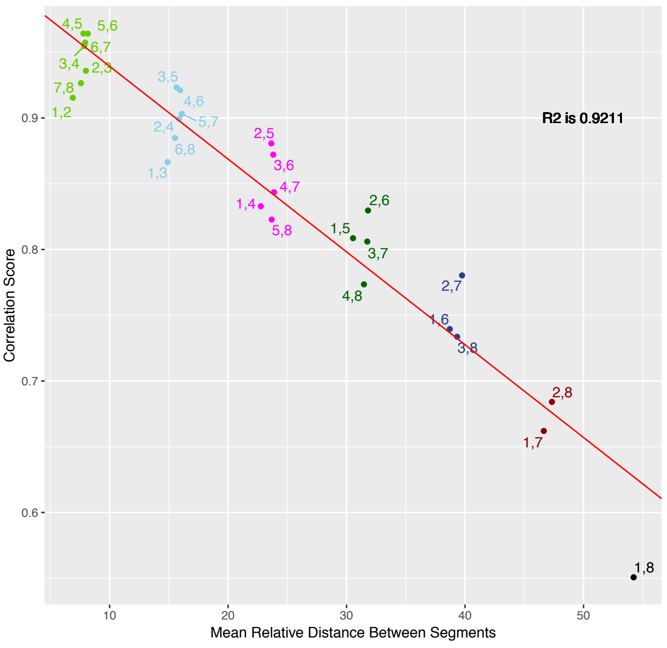

Supplement: Supplementary file 10 — Figure S10. Correlations between relative segment positions within each species. [file EVO-74-1409-s015.pdf]
