# Supplementary material for: Evolution of larval segment position across 12 Drosophila species
Source: Evolution. 2020 Jan 20;74(7):1409–22. doi: 10.1111/evo.13911 (PMC7496318; doi:10.1111/evo.13911)

Figure S11. Correlation coefficient heatmaps for each of the 12 *Drosophila* species. Each graph shows the correlation coefficient between shifts in the relative position (as compared to the across species mean) of each pair of segments within a species. Red indicates high positive correlation and white indicates low positive correlation. Pearson correlation coefficients are printed on each square box. Notice that for most species, the color scheme is lighter at the right edge of the triangle as compared to the bottom edge of the triangle. This is consistent with Figure S10 showing shifts in the position of A8 have a lower correlation with shifts in the position of other segments, regardless of the distance in between any pair of segments.

Supplementary Figure 11

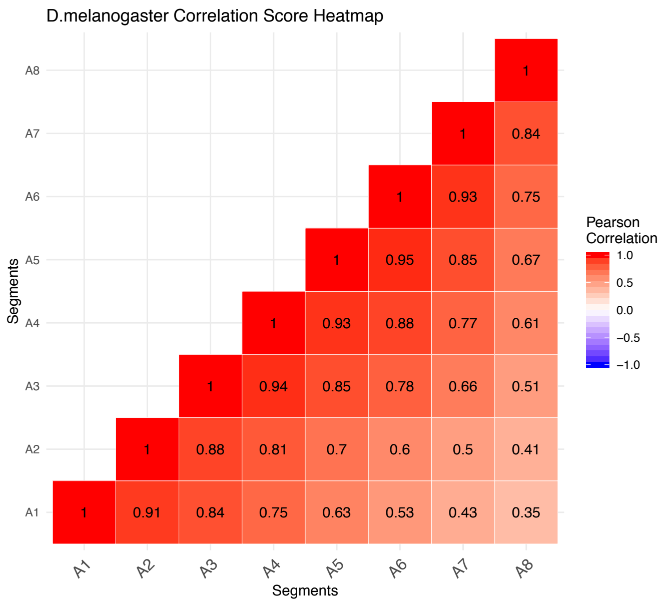

Supplementary Figure 11

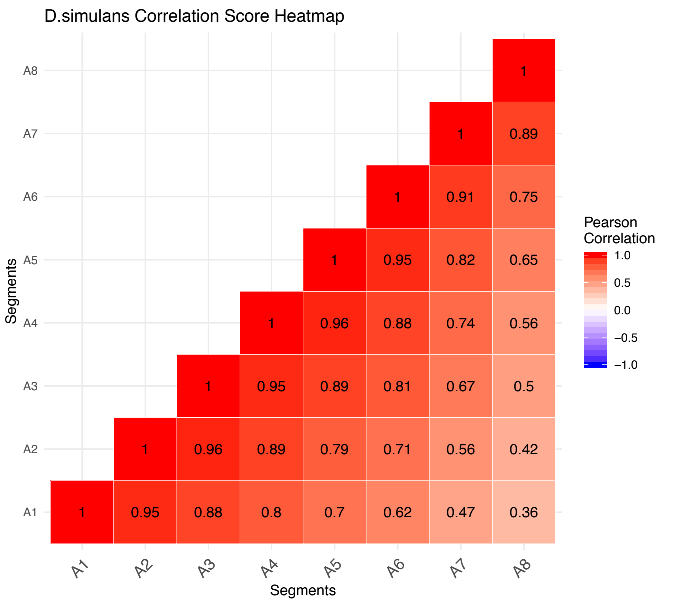

Supplementary Figure 11

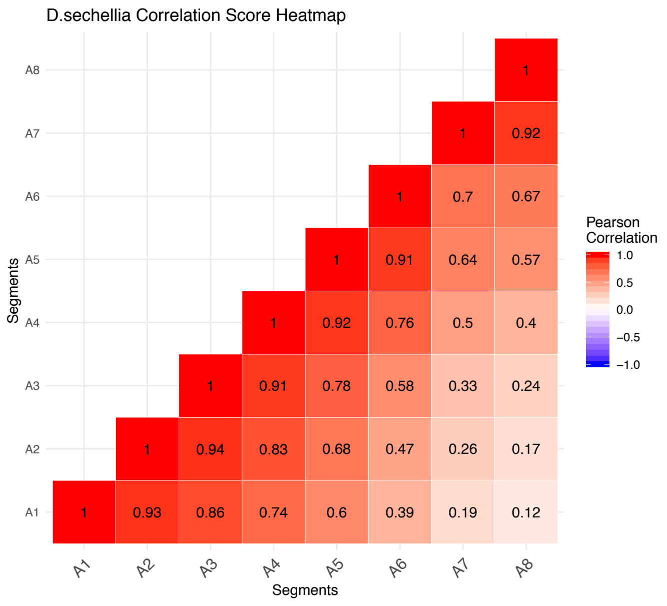

Supplementary Figure 11

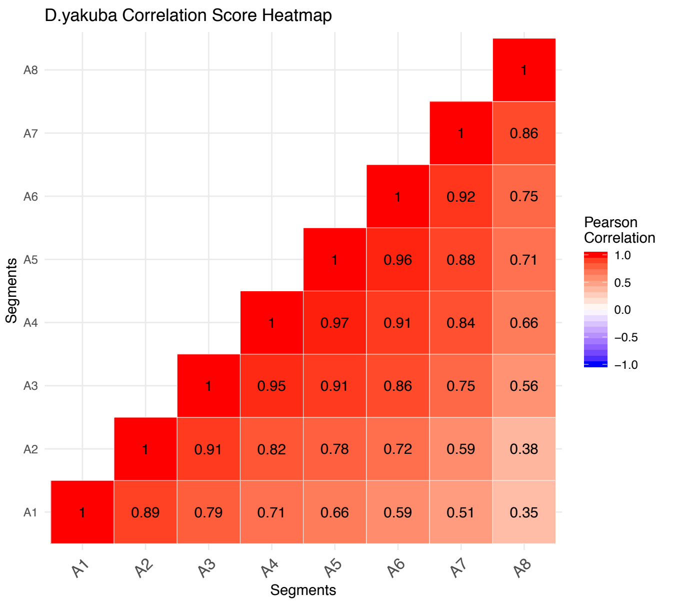

Supplementary Figure 11

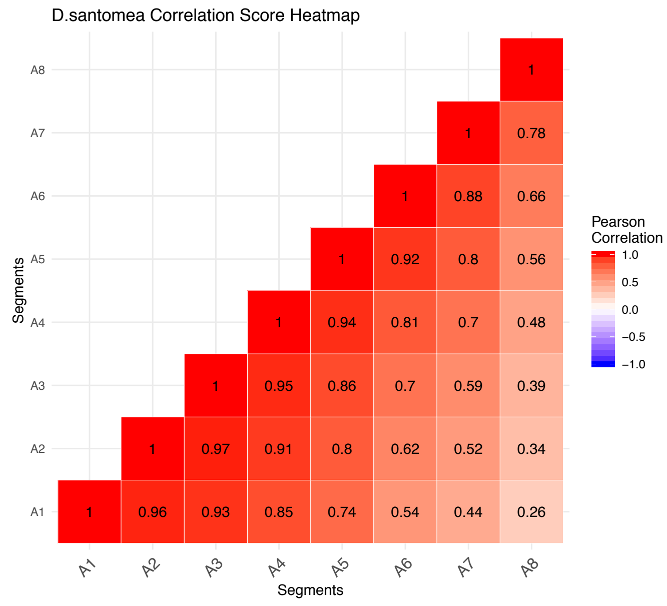

Supplementary Figure 11

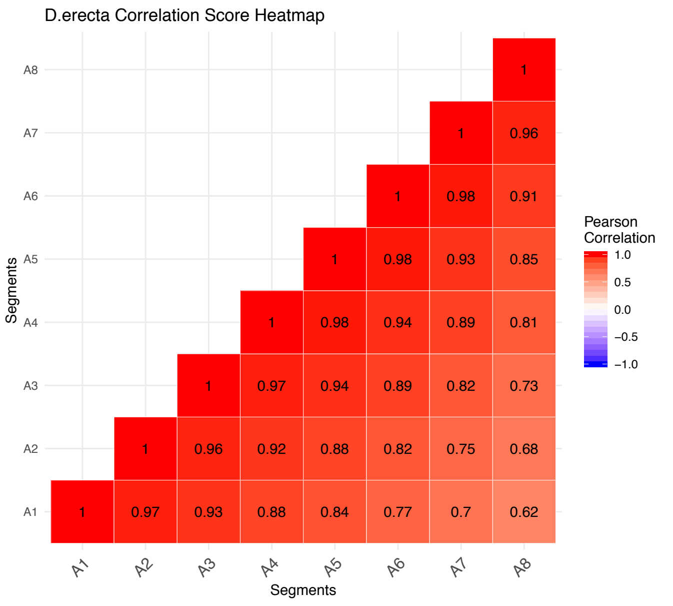

Supplementary Figure 11

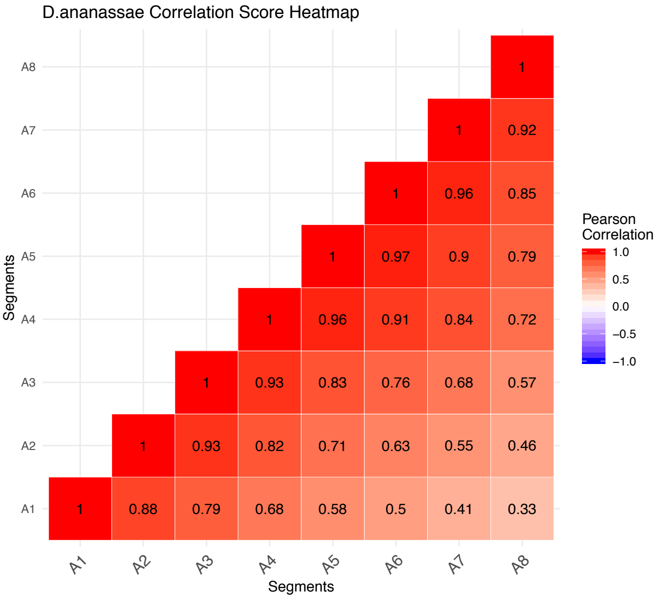

Supplementary Figure 11

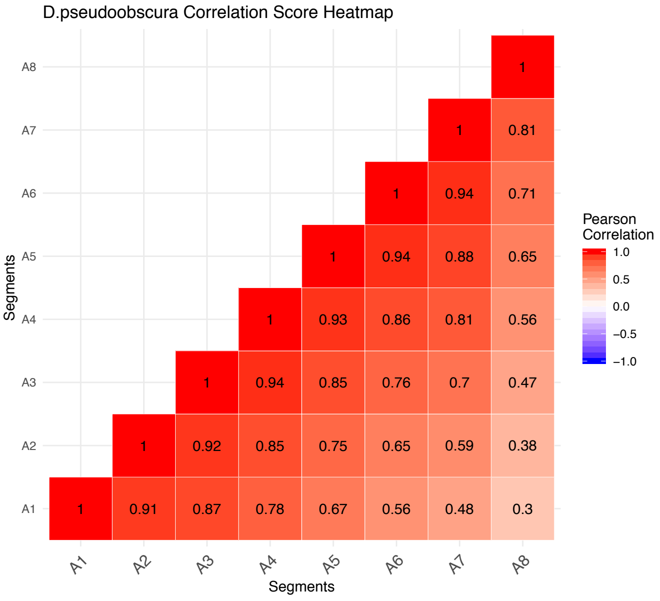

Supplementary Figure 11

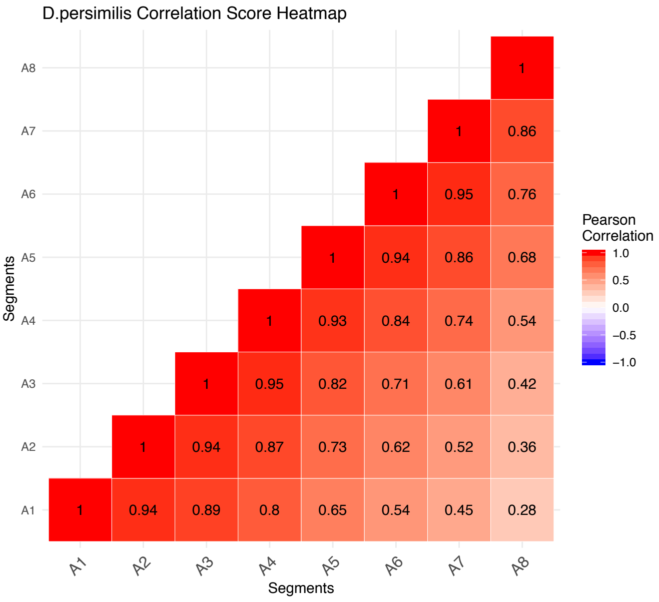

Supplementary Figure 11

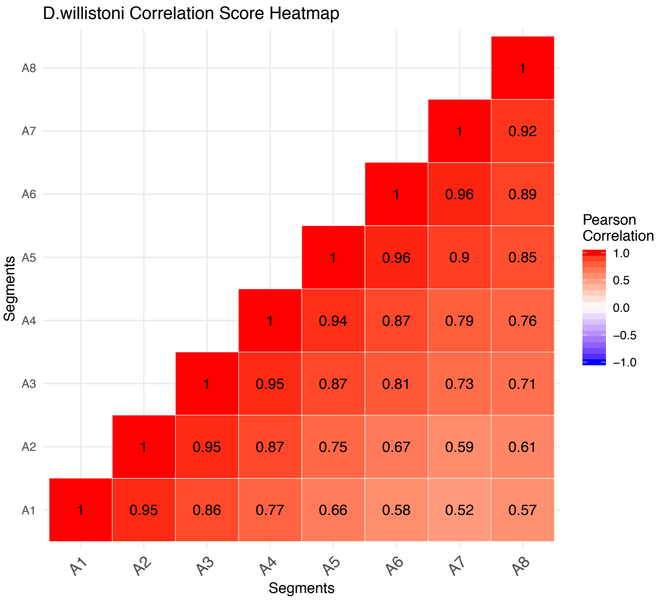

Supplementary Figure 11

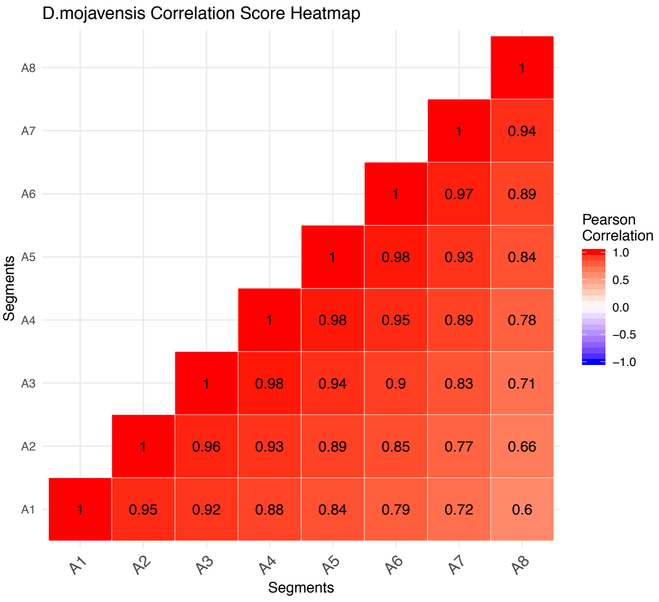

Supplementary Figure 11

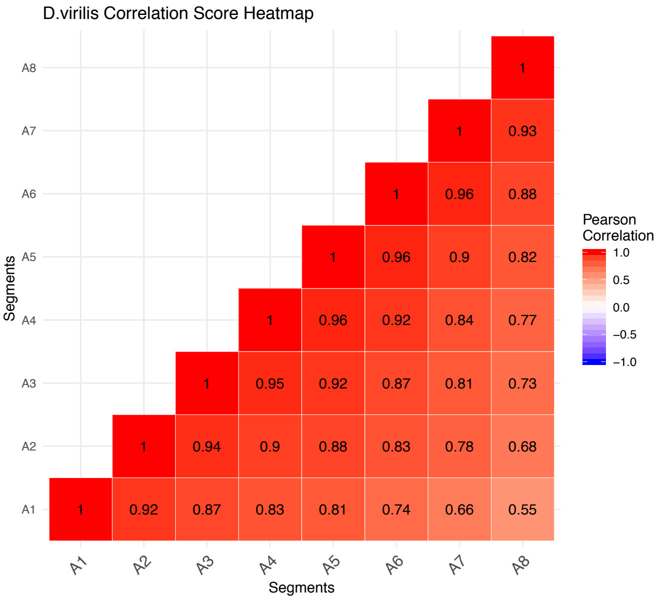

Supplement: Supplementary file 11 — Figure S11. Correlation coefficient heat maps for each of the 12 Drosophila species. [file EVO-74-1409-s016.pdf]
