# Supplementary material for: Evolution of larval segment position across 12 Drosophila species
Source: Evolution. 2020 Jan 20;74(7):1409–22. doi: 10.1111/evo.13911 (PMC7496318; doi:10.1111/evo.13911)

Figure S12. Correlations between segment positions, comparing correlations including segment A8 with all other pairwise comparisons the same number of segments apart. This series of graphs represent the information from Figure 5B, but broken out by how many segments separate the two segments being compared. Each graph has mean correlation coefficient over all species on the y-axis, and mean relative distance in percent larval length over all species on the x-axis. Segments that are paired with A8 are in red. Error bars are 95% confidence intervals.

Supplementary Figure 12

One Segment Apart

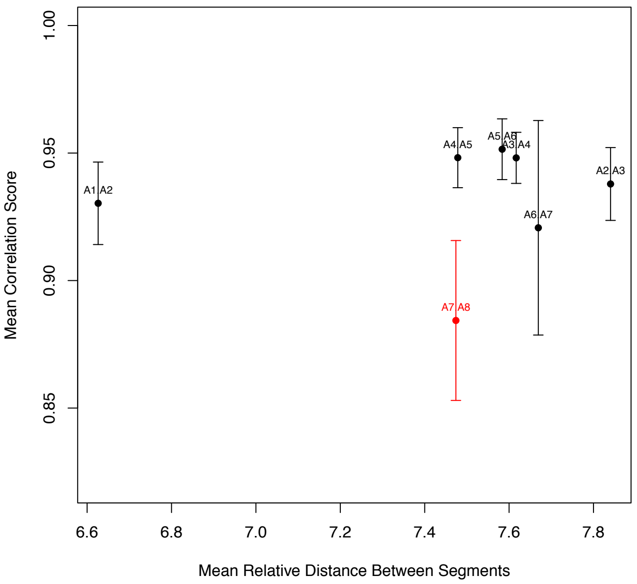

Supplementary Figure 12

Two Segments Apart

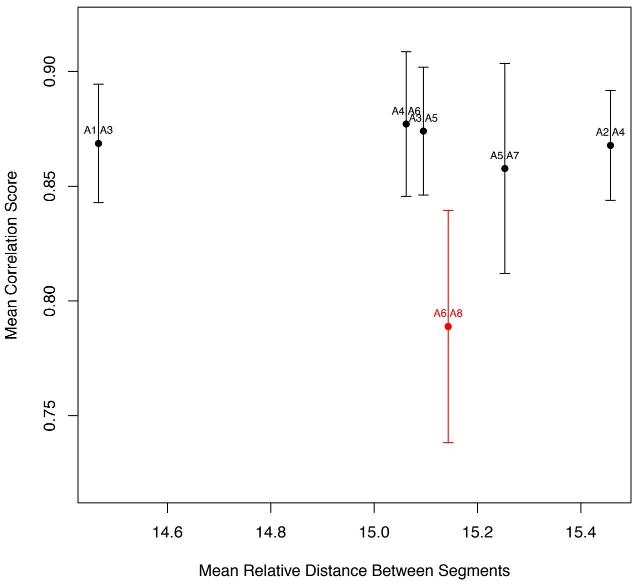

Supplementary Figure 12

Three Segments Apart

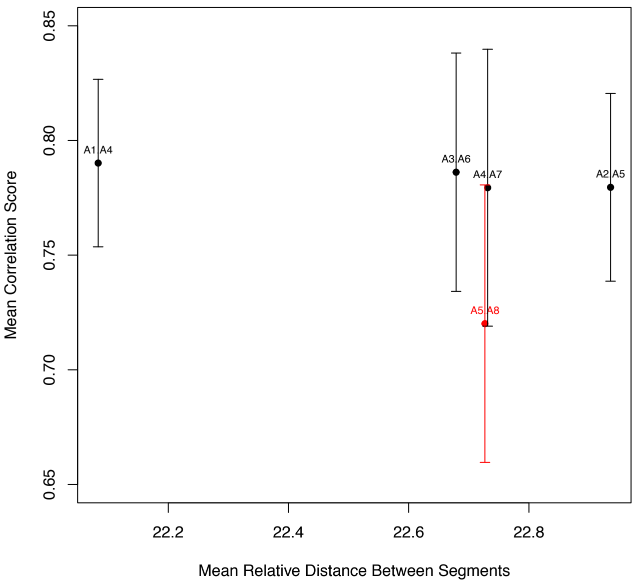

Supplementary Figure 12

Four Segments Apart

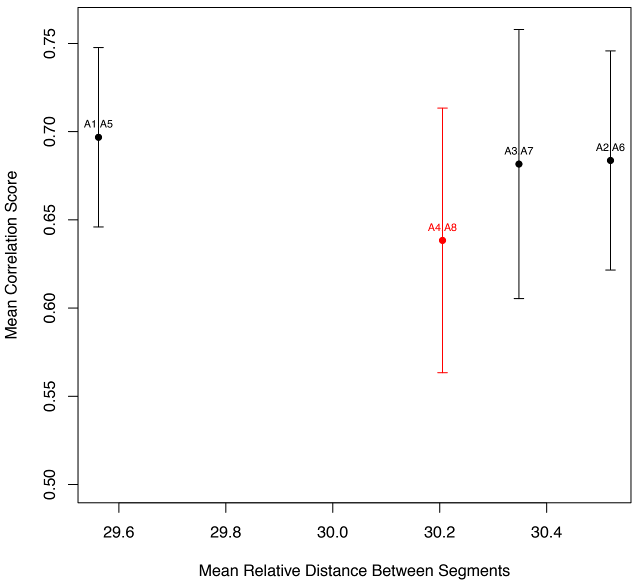

Supplementary Figure 12

Five Segments Apart

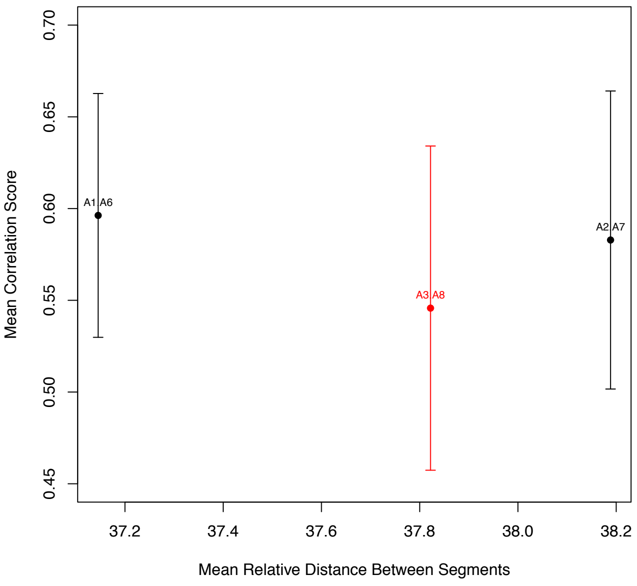

Supplementary Figure 12

Six Segments Apart

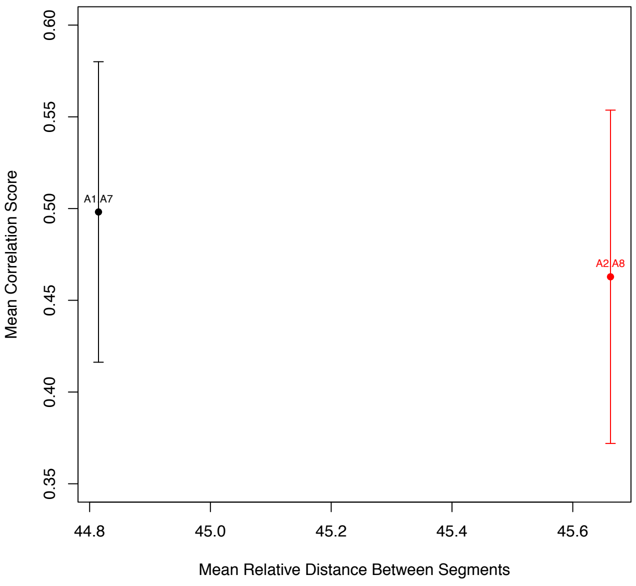

Supplement: Supplementary file 12 — Figure S12. Correlations between segment positions, comparing correlations including segment A8 with all other pairwise comparisons the same number of segments apart. [file EVO-74-1409-s017.pdf]
