# Supplementary material for: Evolution of larval segment position across 12 Drosophila species
Source: Evolution. 2020 Jan 20;74(7):1409–22. doi: 10.1111/evo.13911 (PMC7496318; doi:10.1111/evo.13911)
Supplement: Supplementary file 13 — Figure S13. When all segments are included in position calculations, mean correlation coefficients between neighboring segments are highest, ever so slightly, in the middle of the larva. [file EVO-74-1409-s018.docx]

**Figure S13.** When all segments are included in position calculations, mean correlation coefficients between neighboring segments are highest, ever so slightly, in the middle of the larva. In comparison, when A8+tail is removed from position calculations, correlation between segment position shifts at the posterior of the larvae goes down significantly. This is true for the anterior part of the larva, when h+t are removed from position calculations. y-axis shows mean correlation coefficient over all species and x-axis shows pairs of adjacent segments along the anterior-posterior axis.
