# Supplementary material for: Evolution of larval segment position across 12 Drosophila species
Source: Evolution. 2020 Jan 20;74(7):1409–22. doi: 10.1111/evo.13911 (PMC7496318; doi:10.1111/evo.13911)

Figure S14. This series of graphs are a continuation of Figure S13, as the distance between pairs of segments increase, from two to six segments apart. Notice that removal of h+t overall decreases correlation coefficients more than removal of A8+tail. y-axis shows mean correlation coefficient over all species and x-axis shows pairs of segments, which are two, three, four, five, six segments apart, along the anterior-posterior axis.

Supplementary Figure 14

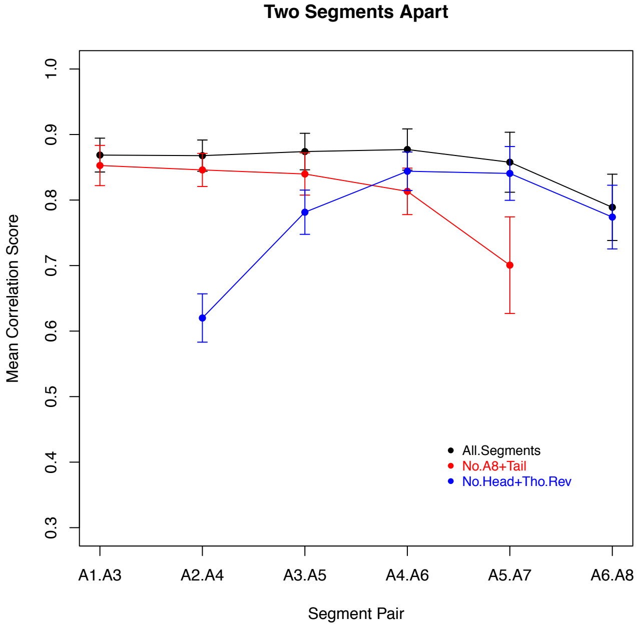

Supplementary Figure 14

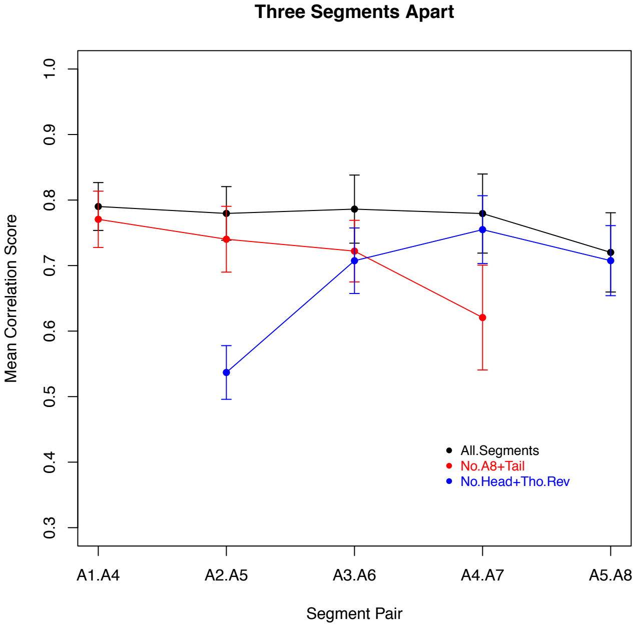

Supplementary Figure 14

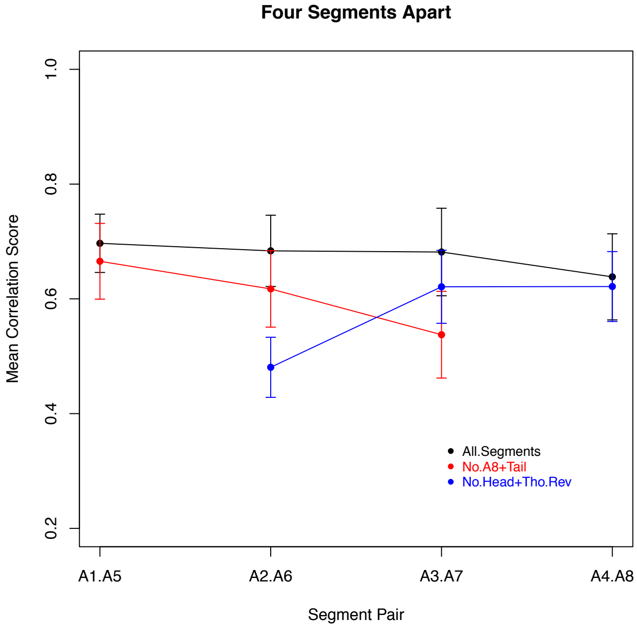

Supplementary Figure 14

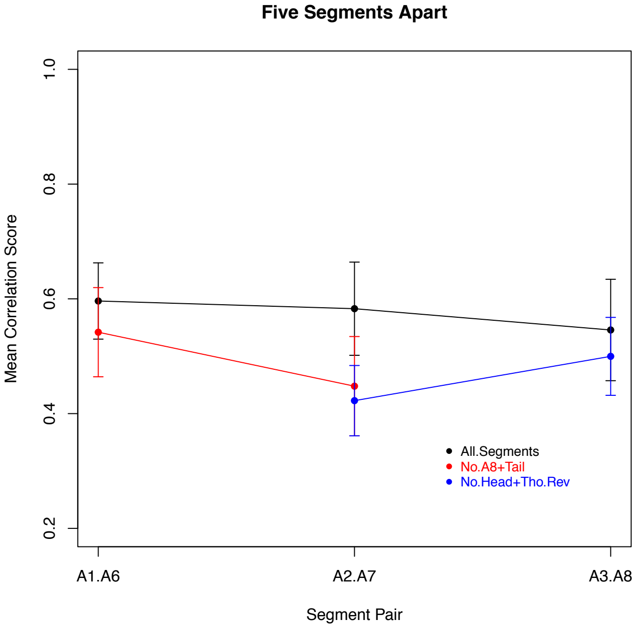

Supplementary Figure 14

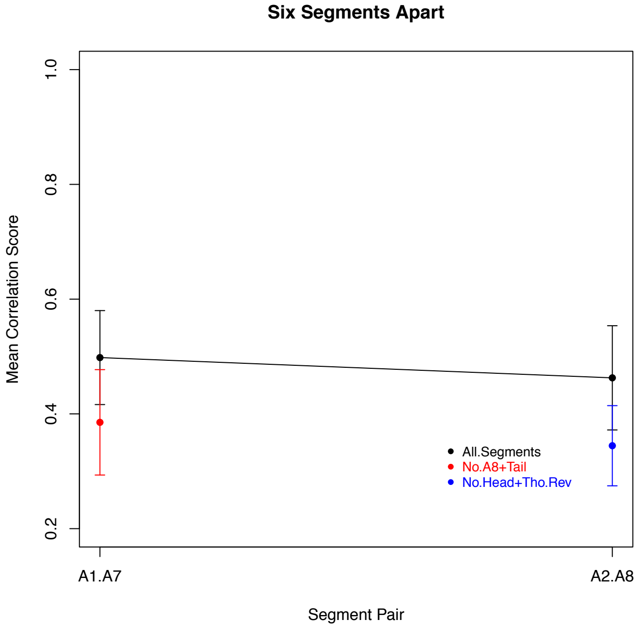

Supplement: Supplementary file 14 — Figure S14. This series of graphs are a continuation of Figure S13, as the distance between pairs of segments increase, from two to six segments apart. [file EVO-74-1409-s019.pdf]
