# Supplementary material for: Evolution of larval segment position across 12 Drosophila species
Source: Evolution. 2020 Jan 20;74(7):1409–22. doi: 10.1111/evo.13911 (PMC7496318; doi:10.1111/evo.13911)

Figure S15. This series of graphs show the data represented in Figure S13 separately for each of the 12 *Drosophila* species. They show, for each species, how correlation coefficients change from the anterior to the posterior of the larvae, when relative segment position was calculated with all segments included (black) versus no A8+tail (red) or no h+t (blue). y-axis shows correlation coefficients in each species, x-axis shows pairs of adjacent segments along the anterior-posterior axis.

Supplementary Figure 15

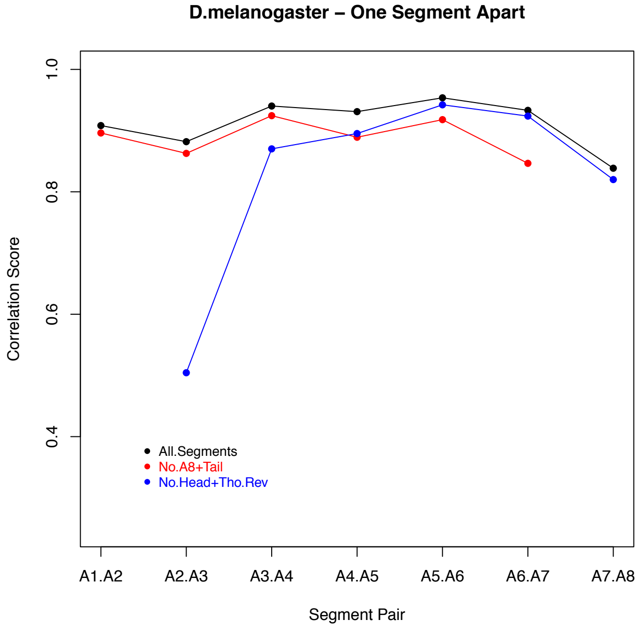

Supplementary Figure 15

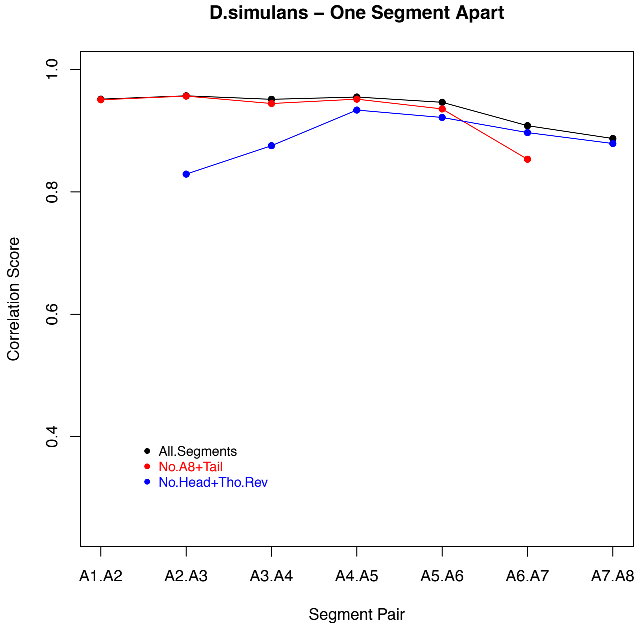

Supplementary Figure 15

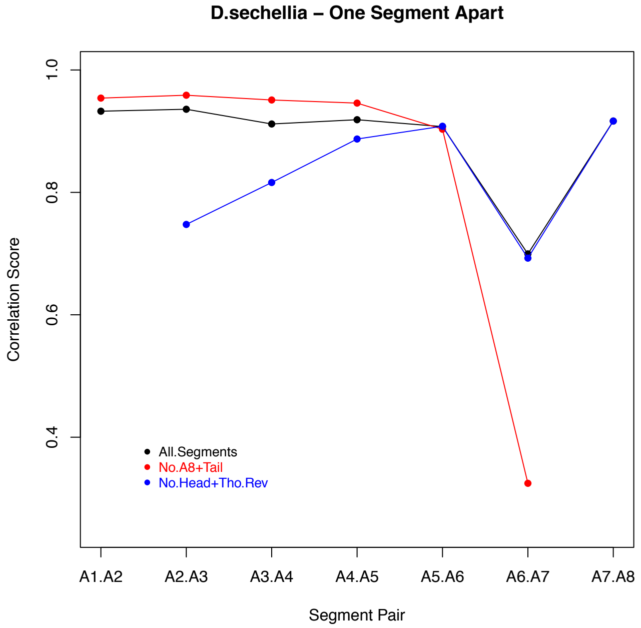

Supplementary Figure 15

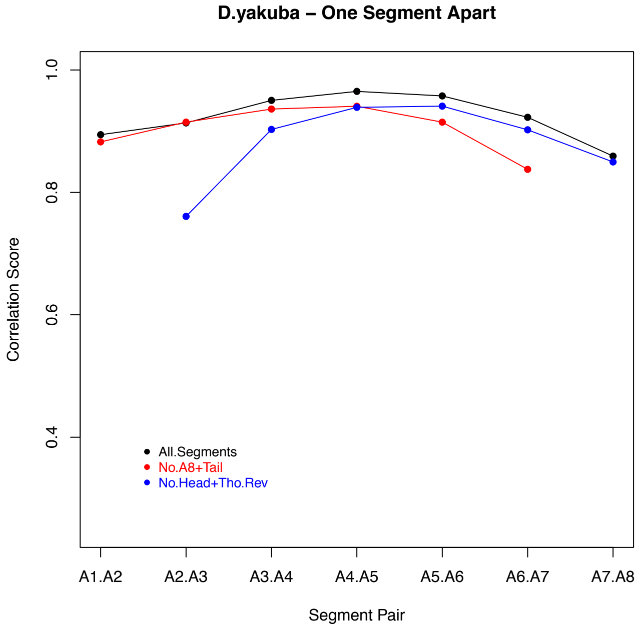

Supplementary Figure 15

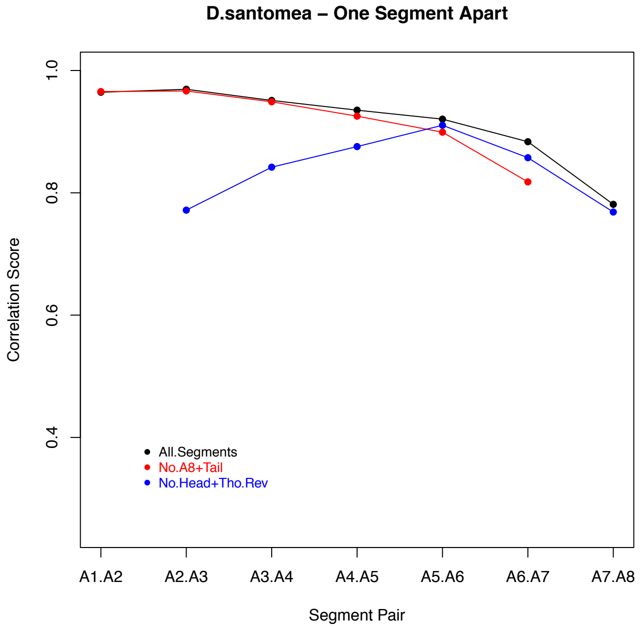

Supplementary Figure 15

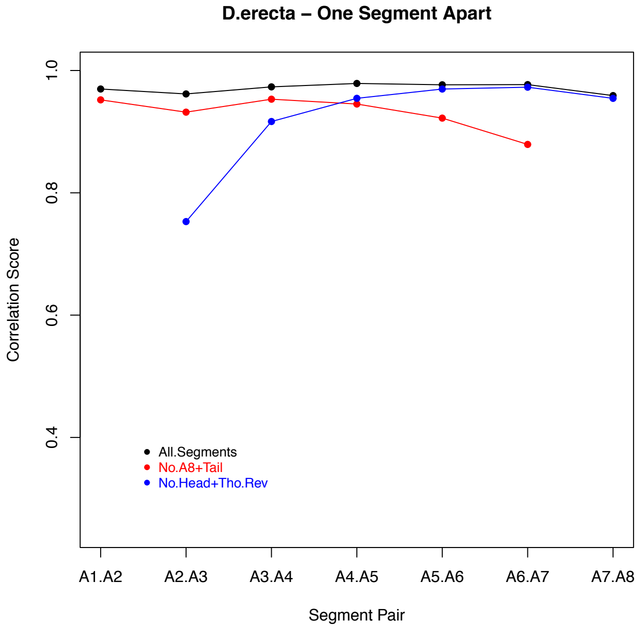

Supplementary Figure 15

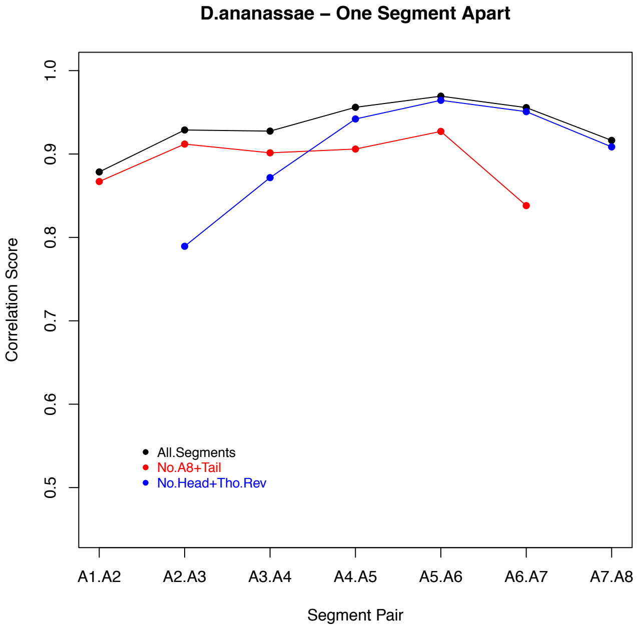

Supplementary Figure 15

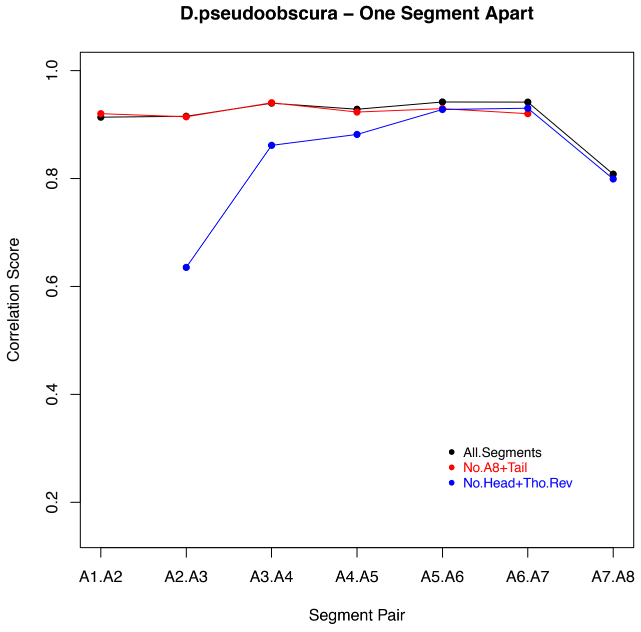

Supplementary Figure 15

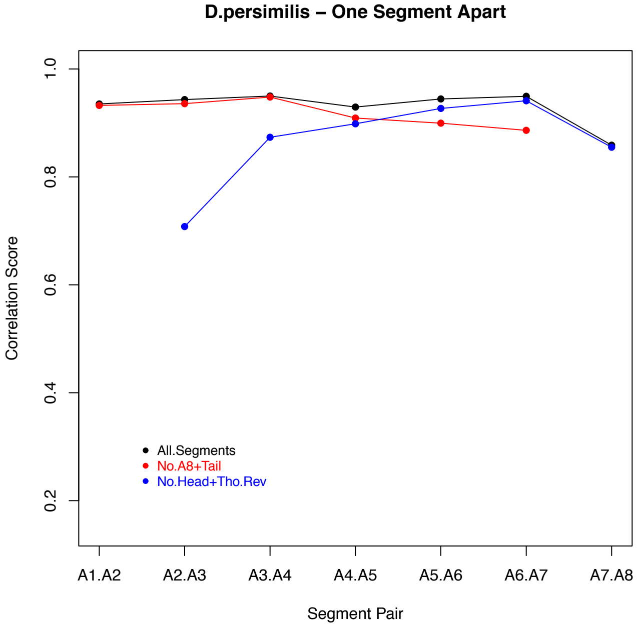

Supplementary Figure 15

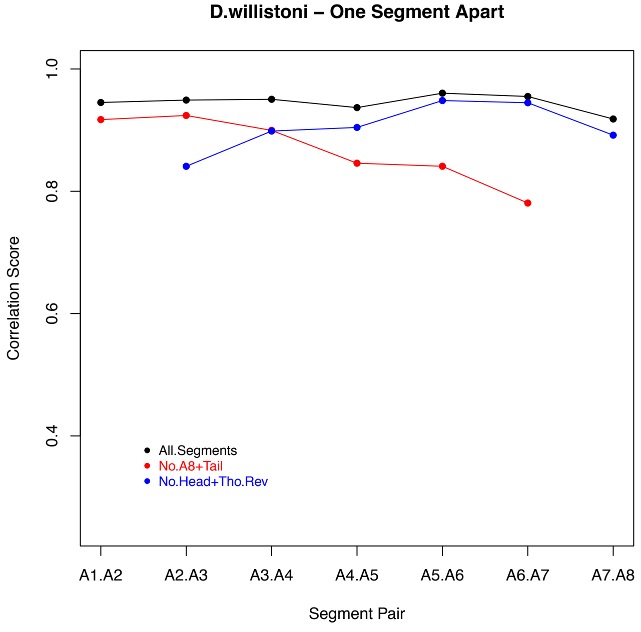

Supplementary Figure 15

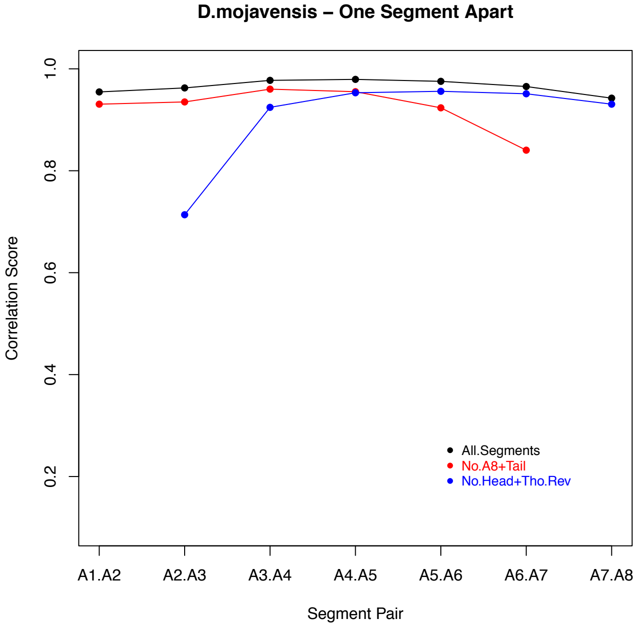

Supplementary Figure 15

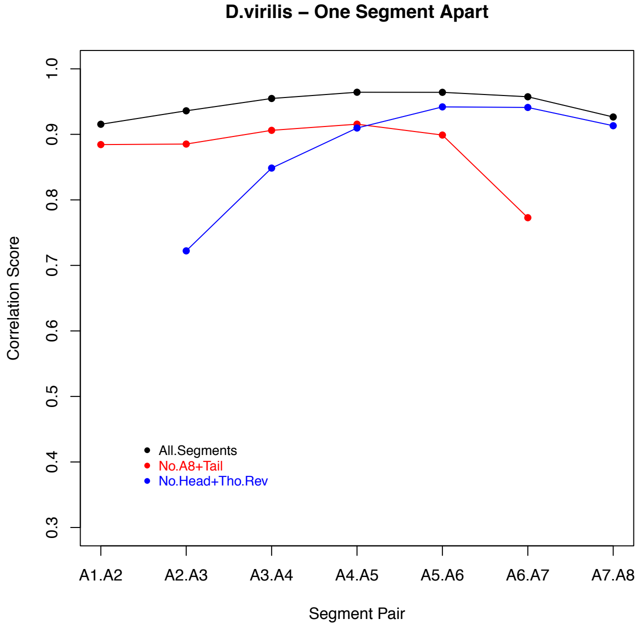

Supplement: Supplementary file 15 — Figure S15. This series of graphs show the data represented in Figure S13 separately for each of the 12 Drosophila species. [file EVO-74-1409-s020.pdf]
