# Supplementary material for: Evolution of larval segment position across 12 Drosophila species
Source: Evolution. 2020 Jan 20;74(7):1409–22. doi: 10.1111/evo.13911 (PMC7496318; doi:10.1111/evo.13911)
Supplement: Supplementary file 16 — Table S1. Experimental methods used for number of flies and days needed in a bottle to control population density of each species. [file EVO-74-1409-s021.docx]

| **Table S1. Experimental methods used for number of flies and days needed in a bottle to control population density of each species.**  **Also includes, for each species, approximate embryonic developmental duration at 20°C and number of minutes needed in the 60°C oven for larvae to become straight.** | | | | | |
| --- | --- | --- | --- | --- | --- |
| **Species Name** | **Number of Male and Female flies per bottle** | **Number of days in bottle before removing adults** | **Number of days from egg to larva at 20°C (hours)** | **Number of minutes at 60° oven** | **Number of larval samples from which measurements were taken for data analysis** |
| D. melanogaster (Ore-R) | 15 each | 4-5 days | ~36 | 50 | 145 |
| D. simulans | 15 each | 4-5 days | ~24 | 50 | 129 |
| D. sechellia | 20 each | 7 days | ~36 | 90 | 120 |
| D. yakuba | 10 each | 4-5 days | ~24 | 50 | 143 |
| D. santomea | 25 each | 7 days | ~36 | 50 | 134 |
| D. erecta | 10 each | 4-5 days | ~24 | 50 | 105 |
| D. ananassae | 20 each | 7 days | ~36 | 50 | 107 |
| D. pseudoobscura | 10 each | 7days | ~36 | 50 | 118 |
| D. persimilis | 15 each | 7 days | ~36 | 50 | 113 |
| D. willistoni | 25 each | 7 days | ~48 | 50 | 112 |
| D. mojavensis | 25 each | 7 days | ~72 | 45 | 110 |
| D. virilis | 15 each | 7 days | ~60 | 90 | 113 |
