# Supplementary material for: Evolution of larval segment position across 12 Drosophila species
Source: Evolution. 2020 Jan 20;74(7):1409–22. doi: 10.1111/evo.13911 (PMC7496318; doi:10.1111/evo.13911)
Supplement: Supplementary file 18 — Table S3. Lists the deviation of the position of each segment in each species from the “across‐species” mean as well as total number of significant segment position changes for each species over all species‐pair comparisons. [file EVO-74-1409-s001.docx]

| **Supplementary Table 3. Lists the deviation of the position of each segment in each species from the “across-species” mean as well as total number of significant segment position changes for each species over all species-pair comparisons.** | | | | | |
| --- | --- | --- | --- | --- | --- |
| species | segment | deviation from the species mean | absolute deviation from the species mean | total (absolute) deviation of mean over all segments | In of all species pair comparisons, number of significant segment position changes for each species |
| Dana | A1 | 0.1568381 | 0.1568381 | 1.74198203 | 56 (13.66%) |
| Dana | A2 | 0.23681895 | 0.23681895 |  |  |
| Dana | A3 | 0.25958437 | 0.25958437 |  |  |
| Dana | A4 | 0.22392065 | 0.22392065 |  |  |
| Dana | A5 | 0.18130454 | 0.18130454 |  |  |
| Dana | A6 | 0.22764646 | 0.22764646 |  |  |
| Dana | A7 | 0.15954071 | 0.15954071 |  |  |
| Dana | A8 | 0.29632824 | 0.29632824 |  |  |
| Dere | A1 | -0.4150311 | 0.41503106 | 8.86873982 | 78 (19.02%) |
| Dere | A2 | -0.5967867 | 0.59678675 |  |  |
| Dere | A3 | -0.8037267 | 0.80372671 |  |  |
| Dere | A4 | -1.0135141 | 1.01351415 |  |  |
| Dere | A5 | -1.1776149 | 1.17761491 |  |  |
| Dere | A6 | -1.4161008 | 1.41610076 |  |  |
| Dere | A7 | -1.7061228 | 1.70612284 |  |  |
| Dere | A8 | -1.7398427 | 1.73984265 |  |  |
| Dmel | A1 | -0.6439966 | 0.64399657 | 3.66891602 | 65 (15.85%) |
| Dmel | A2 | -0.6237096 | 0.62370957 |  |  |
| Dmel | A3 | -0.6214804 | 0.6214804 |  |  |
| Dmel | A4 | -0.3758557 | 0.37585569 |  |  |
| Dmel | A5 | -0.2248037 | 0.22480374 |  |  |
| Dmel | A6 | 0.00472026 | 0.00472026 |  |  |
| Dmel | A7 | 0.51753233 | 0.51753233 |  |  |
| Dmel | A8 | 0.65681745 | 0.65681745 |  |  |
| Dmoj | A1 | -0.0417583 | 0.04175833 | 18.4652117 | 78 (19.02%) |
| Dmoj | A2 | -0.7664274 | 0.76642744 |  |  |
| Dmoj | A3 | -1.2101293 | 1.21012931 |  |  |
| Dmoj | A4 | -1.8972977 | 1.8972977 |  |  |
| Dmoj | A5 | -2.5590608 | 2.55906079 |  |  |
| Dmoj | A6 | -3.28717 | 3.28717002 |  |  |
| Dmoj | A7 | -3.9781228 | 3.97812284 |  |  |
| Dmoj | A8 | -4.7252452 | 4.72524525 |  |  |
| Dper | A1 | 0.94027868 | 0.94027868 | 18.2363837 | 87 (21.22%) |
| Dper | A2 | 1.50173748 | 1.50173748 |  |  |
| Dper | A3 | 1.87890288 | 1.87890288 |  |  |
| Dper | A4 | 2.15942475 | 2.15942475 |  |  |
| Dper | A5 | 2.54855787 | 2.54855787 |  |  |
| Dper | A6 | 2.72579136 | 2.72579136 |  |  |
| Dper | A7 | 3.02886831 | 3.02886831 |  |  |
| Dper | A8 | 3.45282233 | 3.45282233 |  |  |
| Dpse | A1 | -0.0783361 | 0.07833614 | 4.61316168 | 65 (15.85%) |
| Dpse | A2 | 0.06823666 | 0.06823666 |  |  |
| Dpse | A3 | 0.3435009 | 0.3435009 |  |  |
| Dpse | A4 | 0.5554342 | 0.5554342 |  |  |
| Dpse | A5 | 0.67677896 | 0.67677896 |  |  |
| Dpse | A6 | 0.8069178 | 0.8069178 |  |  |
| Dpse | A7 | 1.01891106 | 1.01891106 |  |  |
| Dpse | A8 | 1.06504597 | 1.06504597 |  |  |
| Dsan | A1 | -0.0797325 | 0.07973255 | 2.00922525 | 60 (14.63%) |
| Dsan | A2 | -0.0622402 | 0.0622402 |  |  |
| Dsan | A3 | -0.0621062 | 0.06210624 |  |  |
| Dsan | A4 | -0.1140358 | 0.11403583 |  |  |
| Dsan | A5 | -0.1653498 | 0.1653498 |  |  |
| Dsan | A6 | -0.3448783 | 0.3448783 |  |  |
| Dsan | A7 | -0.4844661 | 0.48446613 |  |  |
| Dsan | A8 | -0.6964162 | 0.69641621 |  |  |
| Dsec | A1 | 1.03255228 | 1.03255228 | 7.58229589 | 78 (19.02%) |
| Dsec | A2 | 1.03628468 | 1.03628468 |  |  |
| Dsec | A3 | 0.93766615 | 0.93766615 |  |  |
| Dsec | A4 | 0.88333109 | 0.88333109 |  |  |
| Dsec | A5 | 0.88232557 | 0.88232557 |  |  |
| Dsec | A6 | 0.79912543 | 0.79912543 |  |  |
| Dsec | A7 | 0.98804382 | 0.98804382 |  |  |
| Dsec | A8 | 1.02296687 | 1.02296687 |  |  |
| Dsim | A1 | 0.32465887 | 0.32465887 | 1.76293065 | 64 (15.61%) |
| Dsim | A2 | 0.02282344 | 0.02282344 |  |  |
| Dsim | A3 | -0.0737156 | 0.07371563 |  |  |
| Dsim | A4 | 0.0538214 | 0.0538214 |  |  |
| Dsim | A5 | 0.22511821 | 0.22511821 |  |  |
| Dsim | A6 | 0.29352272 | 0.29352272 |  |  |
| Dsim | A7 | 0.35652832 | 0.35652832 |  |  |
| Dsim | A8 | 0.41274207 | 0.41274207 |  |  |
| Dvir | A1 | -1.1391903 | 1.13919035 | 5.17973079 | 63 (15.36%) |
| Dvir | A2 | -0.8658731 | 0.86587314 |  |  |
| Dvir | A3 | -0.7026016 | 0.70260155 |  |  |
| Dvir | A4 | -0.4642036 | 0.46420357 |  |  |
| Dvir | A5 | -0.1616191 | 0.16161912 |  |  |
| Dvir | A6 | 0.41118959 | 0.41118959 |  |  |
| Dvir | A7 | 0.66408955 | 0.66408955 |  |  |
| Dvir | A8 | 0.77096392 | 0.77096392 |  |  |
| Dwil | A1 | 0.06595109 | 0.06595109 | 2.19713164 | 58 (14.15%) |
| Dwil | A2 | 0.2999573 | 0.2999573 |  |  |
| Dwil | A3 | 0.52305901 | 0.52305901 |  |  |
| Dwil | A4 | 0.48764061 | 0.48764061 |  |  |
| Dwil | A5 | 0.27636128 | 0.27636128 |  |  |
| Dwil | A6 | 0.19125043 | 0.19125043 |  |  |
| Dwil | A7 | -0.1766943 | 0.17669427 |  |  |
| Dwil | A8 | -0.1762177 | 0.17621765 |  |  |
| Dyak | A1 | -0.0419541 | 0.04195413 | 3.27637951 | 68 (16.58%) |
| Dyak | A2 | -0.1425883 | 0.14258828 |  |  |
| Dyak | A3 | -0.3278426 | 0.32784259 |  |  |
| Dyak | A4 | -0.4455774 | 0.44557742 |  |  |
| Dyak | A5 | -0.5241657 | 0.52416569 |  |  |
| Dyak | A6 | -0.5134567 | 0.51345674 |  |  |
| Dyak | A7 | -0.6486823 | 0.64868228 |  |  |
| Dyak | A8 | -0.6321124 | 0.63211238 |  |  |
